# The Volatile Phytochemistry of Seven Native American Aromatic Medicinal Plants

**DOI:** 10.3390/plants10061061

**Published:** 2021-05-25

**Authors:** Sims K. Lawson, Prabodh Satyal, William N. Setzer

**Affiliations:** 1 Penn State College of Agricultural Sciences, Department of Ecosystem Science and Management, 117 Forest Resources Building, University Park, PA 16802, USA; kirk@psu.edu; 2 Aromatic Plant Research Center, Suite 100, Lehi, UT 84043, USA; psatyal@aromaticplant.org; 3 Department of Chemistry, University of Alabama in Huntsville, Huntsville, AL 35899, USA

**Keywords:** *Agastache*, *Gaultheria*, *Heliopsis*, *Liatris*, *Pycnanthemum*, *Smallanthus*, *Verbena*, essential oil, Native American, ethnobotany

## Abstract

As part of our evaluation of essential oils derived from Native American medicinal plants, we have obtained the essential oils of *Agastache foeniculum* (Pursch) Kuntze (Lamiaceae), *Gaultheria procumbens* L. (Ericaceae), *Heliopsis helianthoides* (L.) Sweet (Asteraceae), *Liatris spicata* (L.) Willd. (Asteraceae), *Pycnanthemum incanum* (L.) Michx. (Lamiaceae), *Smallanthus uvedalia* (L.) Mack. ex Mack. (Asteraceae), and *Verbena hastata* L. (Verbenaceae) by hydrodistillation. The essential oils were analyzed by gas chromatographic techniques. The essential oil of *A. foeniculum* was dominated by estragole (88–93%), while methyl salicylate (91%) dominated the *G. procumbens* essential oil. Germacrene D was the major component in *H. helianthoides* (42%) and *L. spicata* (24%). 1,8-Cineole (31%) and α-terpineol (17%) were the main compounds in *P. incanum* essential oil. The essential oil of *S. uvedalia* showed α-pinene (24%), perillene (15%), and β-caryophyllene (17%) as major components. *Verbena hastata* essential oil was rich in 1-octen-3-ol (up to 29%) and palmitic acid (up to 22%). Four of these essential oils, *H. helianthoides*, *L. spicata*, *P. incanum*, and *V. hastata*, are reported for the first time. Additionally, the enantiomeric distributions of several terpenoid components have been determined.

## 1. Introduction

Plants have been used in traditional medicine since prehistoric times. The therapeutic properties of medicinal plants are generally attributed to secondary metabolites produced by the plants as protection against pathogens and herbivory. As with many other aboriginal peoples, Native North Americans have used plants as medicines throughout their history. Although not as extensively documented as traditional Chinese medicine or Ayurvedic medicine, there are several sources of information regarding Native American ethnopharmacology [1,2,3]. As part of our ongoing investigations of the essential oil compositions of Native American medicinal plants, we have collected and examined seven aromatic medicinal plants growing in the southeastern United States.

*Agastache foeniculum* (Pursch) Kuntze (Lamiaceae) is native to north central United States and southern Canada, but has been recorded in southern Alabama [4]. The plant has been introduced to Europe, particularly the U.K., the Netherlands, and Germany, as an ornamental [5]. Cheyenne and Chippewa Native Americans used an infusion of the leaves of *A. foeniculum* as a cold medicine [2]. Essential oil compositions of *A. foeniculum* have been extensively studied, and the oils are typically dominated by methyl chavicol (estragole) with smaller amounts of (*E*)-anethole [6]. Nonvolatile phytochemicals from *A. foeniculum* include flavonoids (apigenin, quercetin), polyphenolics (rosmarinic acid, caffeic acid), pentacyclic triterpenoids (α-amyrin, β-amyrin), and sterols (campesterol, campestanol, sitosterol, stigmasterol, stigmastanol) [6].

*Gaultheria procumbens* L. (Ericaceae) naturally ranges in eastern North America from Canada, south through Alabama and Georgia [4]. Several native North American tribes used an infusion of *G. procumbens* to treat headaches, colds (Algonquin, Cherokee, Chippewa, Iroquois) or to treat arthritis, rheumatism, and lumbago (Iroquois, Menominee, Ojibwa, Potawatomi) [2]. The Cherokee were known to use a root infusion of *G. procumbens* along with the roots of *Epigaea repens* for chronic indigestion, and they also chewed the leaves as a substitute for chewing tobacco [7]. Much like its Asian relative, *Gaultheria fragrantissima* Wall., the essential oil of *G. procumbens* is dominated by methyl salicylate. Commercial *G. fragrantissima* essential oil (doTERRA International, Pleasant Grove, Utah) has 99.7% methyl salicylate, while methyl salicylate in *G. procumbens* essential oil typically ranges 96.6–99.8% [8,9,10].

*Heliopsis helianthoides* (L.) Sweet (Asteraceae) is native to North America from Saskatchewan, Canada east to the Atlantic coast of Newfoundland and south to the Gulf of Mexico, with the western range extending as far as New Mexico [4,11,12]. There are three subspecies of *H. helianthoides*, ssp. *helianthoides*, generally occurring east of the Mississippi River; ssp. *occidentalis*, found in the Great Plains region; and ssp. *scabra*, which is predominant in the Ozark region (Missouri, Arkansas, and Oklahoma) [11]. The Chippewa took a decoction of the roots of *H. helianthoides* as a stimulant [2]. The Cherokee used the roots in combination with *Scutellaria incana* “for young women”, presumably for menstruation-related discomforts, and sore feet were relieved by soaking in an infusion of what was called “swamp sunflower” [7]. Guaianolide sesquiterpenoids [13], *N*-alkylamides [14], and lignans [15] have been isolated and characterized from *H. helianthoides*.

The natural range of *Liatris spicata* (L.) Willd. (Asteraceae) is the eastern United States and Canada, east of the Mississippi River, from the south along the Gulf of Mexico including southern Alabama and northern Florida areas, north to Ontario and Quebec [4,16,17]. The Cherokee used the plant as an analgesic for backache and limb pains [2]. The guaianolide sesquiterpenoid spicatin has been isolated from the chloroform extract of *L. spicata* [18].

*Pycnanthemum incanum* (L.) Michx. (Lamiaceae) ranges naturally in the eastern United States from the Mississippi River east to the Atlantic coast and from southern Ontario, Canada south to the Gulf of Mexico, though it is primarily found from the Appalachian mountain region beginning in north Georgia north into Ontario, Canada [4,19]. The Cherokee and Choctaw people used the leaves of *P. incanum* externally to treat headaches [2]. Dein and Munafo have characterized the key odorants from *P. incanum* to be β-ionone, myrcene, linalool, and pulegone [20].

The natural range of *Smallanthus uvedalia* (L.) Mack. ex Mack. (Asteraceae) is the southeastern United States, from the Ohio river basin south to the Gulf of Mexico [4]. *Smallanthus uvedalia* was reportedly used internally by Native American Indians for laxative properties as well as a stimulant and also to treat swollen glands, especially mastitis [21]. The Cherokee used a salve of the roots to treat burns and cuts, while the Iroquois took an infusion of the plant for back pain and as an antiemetic [2]. Interestingly, the Cherokee were also reported to have used a tea made from this plant to induce vomiting, though it is unclear which portion was used [7]. A number of germacranolide sesquiterpenoids and *ent*-kaurane diterpenoids have been isolated and characterized from *S. uvedalia* [22]. The leaf essential oils of *S. uvedalia* from several locations in north Alabama have been analyzed previously [23,24].

*Verbena hastata* L. (Verbenaceae) ranges throughout North America [4]. The Cherokee used the plant to treat colds and coughs and to alleviate fevers [2]. The ethanol leaf extract of *V. hastata* has shown antiplasmodial [25], antinociceptive, anti-inflammatory, antipyretic [26], and anti-ulcer activities [27]. The iridoid glycoside hastatoside has been isolated from *V. hastata* [28].

The purpose of this study was to extend our understanding of the volatile phytochemistry of Native American aromatic medicinal plants by examination of the essential oils of these seven plant species, to determine their chemical compositions as well as the enantiomeric distributions of terpenoid constituents.

## 2. Results and Discussion

The essential oils of each species were obtained by hydrodistillation of dried plant material (Table 1).

### 2.1. Agastache foeniculum (Pursch) Kuntze (Lamiaceae)

The aerial parts of three different plant samples of *A. foeniculum* were collected from cultivated plants in Newville, Alabama. Hydrodistillation gave pale yellow essential oils in yields ranging from 1.48% to 2.30% yield. The essential oil compositions are compiled in Table 2.

The *A. foeniculum* essential oil was dominated by the phenylpropanoid methyl chavicol (=estragole). There are apparently five different chemotypes of *A. foeniculum* based on essential oil chemical profiles: (1) methyl chavicol, (2) spathulenol/bornyl acetate, (3) γ-cadinene/α-cadinol, (4) limonene, and (5) isomenthone [33,34]. Most *A. foeniculum* essential oils belong to the methyl chavicol chemotype, however [33,35,36,37,38,39,40,41,42].

Methyl chavicol contributes to the anise-like aroma of *A. foeniculum* and has shown anti-inflammatory and anti-edematogenic [43,44], cytotoxic [45], and antimicrobial activities against phytopathogens [46,47]. Unfortunately, however, methyl chavicol has also shown genotoxic and carcinogenic activities [48,49].

### 2.2. Gaultheria procumbens L. (Ericaceae)

The leaf essential oil of *G. procumbens* was obtained in 4.25% yield and the major component was methyl salicylate (91.1%) (Table 3). Methyl salicylate is the dominating component in *G. procumbens* essential oil regardless of geographical location of cultivation, ranging in concentration from 61.14% to 99.96% [8,9,10,50,51,52].

The major component of *G. procumbens* essential oil, methyl salicylate, is well-known as an anti-inflammatory, antipyretic, analgesic agent [53], and accounts for the traditional use of the herb by Native Americans. Methyl salicylate is a common flavoring and fragrance ingredient in cosmetics, shampoos, toilet soaps, and other toiletries, however, it is also a potentially hazardous intoxicant [54,55,56].

### 2.3. Heliopsis helianthoides (L.) Sweet (Asteraceae)

The essential oil from the aerial parts of *H. helianthoides* was obtained in 0.95% yield. The major component in the essential oil was germacrene D (42.4%), with a lesser amount of 4-vinylguaicol (5.5%) (Table 4). As far as we are aware, this is the first report on the chemical composition of *H. helianthoides* essential oil.

Although not necessarily a phytochemical marker of the family, germacrene D has been found to be a major component in several members of the Asteraceae. For example, germacrene D is the dominant compound in the essential oils of *Centaurea hadimensis* Wagenitz, K. Ertugrul & H. Dural (44.3%) [57], *Centaurea pseudoscabiosa* Boiss. & Buhse (36.0%) [57], *Eupatorium cannabinum* L. (33.5%) [58], *Polymnia canadensis* L. (63.6%) [23], *Rudbeckia fulgida* Aiton (30.1%) [59], *Rudbeckia hirta* L. (23.6%) [59], *Solidago canadensis* L. (64.1%) [60], *Symphyotrichum novae-angliae* (L.) G.L. Nesom (25.5%) [59], *Verbesina macrophylla* (Cass.) F.S. Blake (37.3%) [61], *Verbesina turbacensis* Kunth (36.9%) [62], and *Liatris spicata* (23.7%, this work, see below). Germacrene D has shown antimicrobial and cytotoxic activities [63,64].

### 2.4. Liatris spicata (L.) Willd. (Asteraceae)

The essential oil composition of *L. spicata* essential oil is presented in Table 5. The major components were germacrene D (23.7%), myrcene (13.7%), α-pinene (8.1%), and caryophyllene oxide (5.9%). Apparently, there have been no previous reports on the essential oil composition of *L. spicata*.

### 2.5. Pycnanthemum incanum (L.) Michx. (Lamiaceae)

Table 6 shows the chemical composition of the essential oil from the aerial parts of *P. incanum* growing wild in South Carolina. The essential oil was rich in oxygenated monoterpenoids, including 1,8-cineole (30.7%), α-terpineol (16.9%), borneol (8.2%), and *cis*-sabinene hydrate (5.6%). The sesquiterpene hydrocarbons (*E*)-β-caryophyllene (11.0%), and germacrene D (5.0%) were also relatively abundant. To our knowledge, this is the first reported analysis of *P. incanum* essential oil. Volatiles obtained from a diethyl ether extract have been analyzed by gas chromatography-olfactometry to determine the key odorants [20]. Although the percentages of the volatiles were not reported, the enantiomeric distributions of several components were determined (Table 6). 1-Octen-3-ol, isomenthone, α-terpineol, and pulegone showed comparable enantiomeric distributions between *P. incanum* essential oil and the volatiles from the previously published diethyl ether extract [20]. Concentrations of α-pinene, linalool, and menthol were too low in this current study to obtain reliable enantiomeric distributions for comparison.

### 2.6. Smallanthus uvedalia (L.) Mack. ex Mack. (Asteraceae)

The essential oil composition of *S. uvedalia* from South Carolina is summarized in Table 7. The major components of *S. uvedalia* essential oil were α-pinene (23.9%), (*E*)-β-caryophyllene (16.9%), perillene (14.5%), germacrene D (12.2%), and limonene (6.1%). In comparison, *S. uvedalia* from northern Alabama (collected in September 2018) contained α-pinene (62.6%), limonene (11.4%), and β-pinene (6.0%), with lesser concentrations of (*E*)-β-caryophyllene (3.8%) [24]. Neither perillene nor germacrene D were observed in this northern Alabama sample. In contrast, two *S. uvedalia* samples collected in February, 2016, from northern Alabama were rich in (*E*)-β-caryophyllene (24.5% and 16.5%) and caryophyllene oxide (19.8% and 14.2%) [23]. α-Pinene concentrations were low (1.3% and 0.0%) and neither perillene nor germacrene D were observed. The differences in compositions in *S. uvedalia* may be attributed to geographical location and/or seasonal variation.

There does not seem to be a trend in the major enantiomers for essential oils of the Asteraceae (see Supplementary Table S1). For example, (+)-α-pinene was the only enantiomer observed in *Erechtites hieracifolia* (L.) Raf. [65], but (–)-α-pinene predominated in *S. uvedalia* (this work). Likewise, (+)-β-pinene was the only enantiomer in *Coreopsis capillacea* Kunth (syn. *C. triloba* S.F. Blake) [66], while (–)-β-pinene was the dominant enantiomer in *Achillea ligustica* All. [67]. (+)-Limonene was the dominant enantiomer in *S. uvedalia* (this work) and *Solidago canadensis* L. [68], whereas the (–)-enantiomer dominated *E. hieracifolia* [65] and *C. capillacea* [66].

### 2.7. Verbena hastata L. (Verbenaceae)

Three different specimens of *V. hastata* were collected and investigated (Table 8). Although collected from the same general location on the same day, the essential oils showed notable quantitative differences in their compositions. For example, the concentration of 1-octen-3-ol ranged from 2.4% to 29.1%, nonanal ranged from 1.8% to 11.1%, palmitic acid 8.5–21.6%, 1-octadecanol 2.8–14.0%, and phytol 5.2–12.6%. The enantiomeric distributions of linalool, α-terpineol, (*E*)-β-ionone, and (*E*)-nerolidol were the same for the three samples, however. As far as we know, this is the first report on the essential oil composition of *V. hastata*.

There are few *Verbena* essential oil compositions to compare. However, several *Verbena officinalis* L. essential oils have been reported, and these samples also show wide variation in composition. The major components in *V. officinalis* essential oil from Morocco were spathulenol (10.8%), limonene (7.5%), 1,8-cineole (7.5%), caryophyllene oxide (7.3%), and *ar*-curcumene (6.0%) [69]. In contrast, the essential oil of *V. officinalis* from Italy was rich in geranial (45.5%) and isobornyl formate (41.4%) [70]. *Verbena officinalis* from Algeria, on the other hand, showed limonene (17.7%), geranial (14.8%), carvone (14.2%), and caryophyllene oxide (12.4%) as major components [71].

The (–)-enantiomer of 1-octen-3-ol was the only stereoisomer observed in *V. hastata* essential oils as it was in *P. incanum* essential oil (above). Notably, (–)-1-octen-3-ol is the major enantiomer, generally greater than 97%, in mushrooms [72], and is the stereoisomer responsible for mushroom odor [73]. Interestingly, although both enantiomers and the racemic mixture of 1-octen-3-ol attract mosquitoes, the (–)-enantiomer attracted more mosquito species [74].

A racemic mixture was observed for α-terpineol, but there was a higher concentration of (–)-linalool over (+)-linalool in *V. hastata*. (–)-Linalool also dominated in the essential oil of *Lantana camara* L. (Verbenaceae) from Madagascar [75]. In contrast, linalool in the essential oil of *Lippia alba* (Mill.) N.E. Brown (Verbenaceae) from Uruguay was dominated by the (+)-enantiomer [76].

## 3. Materials and Methods

### 3.1. Plant Material

The aerial parts of *A. foeniculum*, *G. procumbens*, and *H. helianthoides* were obtained from plants cultivated in at Kirkland Gardens, in Newville, Alabama (Table 1). The cultivated plants were started from commercially available seeds (*A. foeniculum*, Homegrown Seed Company, and *H. helianthioides*, Prairie Moon Nursery), tubers (*L. spicata*, Wal-Mart), or young plants (*G. procumbens*, The Home Depot). All the plants were grown in full sun, except the *G. procumbens*, which was located in a partially shaded location (4 h/day average sunlight), and all were watered at least once a week. The plants were cultivated directly in the ground, which was clayey-loamy sand, which was amended with composted chicken manure, worm castings, kelp meal, and bone meal at time of planting. *Pycnanthemum incanum* and *S. uvedalia* were collected in the wild from a fully shaded forest understory roadside location near a small waterfall in northern South Carolina. The plants were located beside highway 276 near the North Carolina–South Carolina border (see Table 1). The soil was a thick loam with a lot of leaf litter. *Verbena hastata* was collected in the wild near a disturbed fence-line area with full sun between a bovine field and a paved county road in rural Newville, Alabama (Table 1). Specimens of each plant were collected during the flowering phase (Table 1). Voucher specimens of *P. incanum* (SKL83120), *S. uvedalia* (SKL31820), and *V. hastata* (SKL51321) were deposited in the University of Alabama in Huntsville Herbarium (HALA); cultivated plants were not vouchered. For each species, the plant material was air-dried in the laboratory (around 23 °C) for 10 days. The dried plant materials of each species were chopped and hydrodistilled using a Likens–Nickerson apparatus with continuous extraction with dichloromethane for 4 h. The dichloromethane was evaporated using a stream of dry nitrogen to give the essential oils (Table 1).

### 3.2. Gas Chromatographic Analysis

The essential oils were analyzed by gas chromatography-mass spectrometry (GC-MS), gas chromatography-flame ionization detection (GC-FID), and chiral GC-MS as previously described [77].

GC-MS: Shimadzu GCMS-QP2010 Ultra, electron impact (EI) mode (electron energy = 70 eV), scan range = 40–400 atomic mass units, scan rate = 3.0 scans/s, and GC-MS solution software; ZB-5ms fused silica capillary column (30 m length × 0.25 mm inner diameter) with a (5% phenyl)-polymethylsiloxane stationary phase and a film thickness of 0.25 μm; He carrier gas with a column head pressure of 552 kPa and flow rate of 1.37 mL/min; injector temperature = 250 °C, ion source temperature = 200 °C; GC oven temperature 50–260 °C (2 °C/min), 1-μL injection of 5% solution of EO in dichloromethane (split mode, 30:1). The essential oil components were identified by MS fragmentation, and retention indices compared with those in the databases [29,30,31,32].

GC-FID: Shimadzu GC 2010 equipped with flame ionization detector, a split/splitless injector, and Shimadzu autosampler AOC-20i, with a ZB-5 capillary column (30 m length × 0.25 mm inner diameter) with a (5% phenyl)-polymethylsiloxane stationary phase and a film thickness of 0.25 μm; oven temperature was programmed the same as above for GC-MS; injector temperature = 250 °C, detector temperature = 280 °C, N_2_ carrier gas, and flow rate = 1.0 mL/min. The composition percentages were calculated from raw peak areas without standardization.

Chiral GC-MS: Shimadzu GCMS-QP2010S, EI mode (electron energy = 70 eV) with scan range of 40–400 amu and scan rate of 3.0 scans/s; Restek B-Dex 325 capillary column (30 m × 0.25 mm ID × 0.25 μm film); GC oven temperature program, 50–120 °C (1.5 °C/min), 120–200 °C (2 °C/min), and kept at 200 °C for 5 min; He carrier gas, flow rate = 1.8 mL/min; 0.1-μL injection of 3% solution of EO in dichloromethane (split mode, 1:45). The monoterpenoid enantiomers were identified by comparison of retention times with authentic samples obtained from Sigma-Aldrich (Milwaukee, WI, USA). Relative enantiomer percentages were determined based on peak areas. Chiral GC-MS chromatograms are available as Supplementary Figures S1–S7.

## 4. Conclusions

This report presented the essential oil compositions of seven aromatic medicinal plants used by Native Americans. Four of these essential oils, *Heliopsis helianthoides*, *Liatris spicata*, *Pycnanthemum incanum*, and *Verbena hastata*, were reported for the first time. Additionally, the enantiomeric distributions of several terpenoid components have been determined. The chemical compositions presented add to our knowledge of the phytochemistry of the medicinal plants.

## Figures and Tables

**Table 1:** Collection and hydrodistillation details of seven Native American medicinal plants ^a^.

Plant Species	Collection Site (Date)	Mass of Plant Material	Essential Oil Yield
*Agastache foeniculum* #1	Cultivated, Kirkland Gardens, 31° 26″ 27′ N, 85° 21″ 31′ W (5 May 2020)	24.78 g dried aerial parts	367.1 mg (1.48%) pale yellow essential oil
*Agastache foeniculum* #2	Cultivated, Kirkland Gardens, 31° 26″ 27′ N, 85° 21″ 31′ W (5 May 2020)	28.22 g dried aerial parts	587.7 mg (2.08%) pale yellow essential oil
*Agastache foeniculum* #3	Cultivated, Kirkland Gardens, 31° 26″ 27′ N, 85° 21″ 31′ W (5 May 2020)	13.77 g dried aerial parts	316.6 mg (2.30%) pale yellow essential oil
*Gaultheria procumbens*	Cultivated, Kirkland Gardens, 31° 26″ 27′ N, 85° 21″ 31′ W (10 June 2020)	3.01 g dried leaves	127.8 mg (4.25%) pale yellow essential oil
*Heliopsis helianthoides*	Cultivated, Kirkland Gardens, 31° 26″ 27′ N, 85° 21″ 31′ W (24 July 2020)	27.70 g dried aerial parts	263.6 mg (0.95%) pale yellow essential oil
*Liatris spicata*	Cultivated, Kirkland Gardens, 31° 26″ 27′ N, 85° 21″ 31′ W (24 July 2020)	31.90 g dried aerial parts	133.1 mg (0.420%) pale yellow essential oil
*Pycnanthemum incanum*	Wild-growing, South Carolina, 35° 06’ 08.1″ N, 82° 36’ 44.4″ W (31 August 2020)	9.82 g dried aerial parts	168.1 mg (1.71%) pale yellow essential oil
*Smallanthus uvedalia*	Wild-growing, South Carolina, 35° 06’ 08.1″ N, 82° 36’ 44.4″ W (31 August 2020)	90.77 g dried aerial parts	369.7 mg (0.41%) yellow essential oil
*Verbena hastata* #1	Wild-growing, Newville, Alabama, 31° 26″ 27′ N, 85° 21″ 31′ W (25 May 2020)	24.61 g dried aerial parts	134.2 mg (0.55%) pale yellow essential oil
*Verbena hastata* #2	Wild-growing, Newville, Alabama, 31° 26″ 27′ N, 85° 21″ 31′ W (25 May 2020)	28.50 g dried aerial parts	62.2 mg (0.22%) pale yellow essential oil
*Verbena hastata* #3	Wild-growing, Newville, Alabama, 31° 26″ 27′ N, 85° 21″ 31′ W (25 May 2020)	36.22 g dried aerial parts	214.2 mg (0.59%) pale yellow essential oil

^a^ The volatile materials reported in this research were obtained by hydrodistillation with continuous solvent extraction using a Likens–Nickerson apparatus. Many researchers consider these products not to be true “essential oils”, but rather “volatile fractions”.

**Table 2:** Essential oil composition of three samples of anise fennel (*Agastache foeniculum*) cultivated in south Alabama.

RI_calc_	RI_db_	Compound	Percent Composition		
			#1	#2	#3
924	927	α-Thujene	tr	tr	---
932	933	α-Pinene	tr	tr	tr
963	964	Benzaldehyde	tr	0.1	0.1
971	972	Sabinene	tr	tr	tr
971	974	Hexanoic acid	---	---	tr
975	974	1-Octen-3-ol	0.3	0.7	0.6
983	984	3-Octanone	0.1	0.2	0.2
987	989	Myrcene	0.1	0.1	tr
1016	1018	α-Terpinene	0.1	---	---
1024	1024	*p*-Cymene	0.6	tr	---
1028	1030	Limonene	1.5	4.9	2.9
1030	1033	Benzyl alcohol	---	0.1	0.1
1030	1031	β-Phellandrene	tr	tr	tr
1032	1032	1,8-Cineole	tr	---	---
1057	1057	γ-Terpinene	0.1	---	---
1066	1068	Acetophenone	tr	---	---
1069	1069	*cis*-Sabinene hydrate	tr	---	---
1097	1099	Linalool	0.1	tr	tr
1104	1107	1-Octen-3-yl acetate	0.1	0.2	0.1
1198	1197	Methyl chavicol (=Estragole)	93.2	88.4	91.5
1213	1217	Coumaran	---	tr	tr
1248	1250	Chavicol	0.1	0.2	0.3
1282	1285	(*E*)-Anethole	---	tr	tr
1287	1289	Thymol	0.9	0.1	0.1
1295	1296	Carvacrol	0.1	tr	tr
1309	1309	4-Vinylguaiacol	---	tr	tr
1333	1334	Bicycloelemene	---	tr	tr
1355	1356	Eugenol	0.1	0.1	0.2
1362	1362	Chavibetol	0.7	2.3	0.7
1379	1379	(*E*)-β-Damascenone	---	tr	tr
1384	1382	β-Bourbonene	---	tr	tr
1390	1390	*trans*-β-Elemene	---	tr	tr
1392	1392	(*Z*)-Jasmone	---	tr	0.1
1401	1403	Methyl eugenol	tr	0.1	tr
1417	1417	(*E*)-β-Caryophyllene	1.2	1.6	1.9
1452	1453	α-Humulene	tr	0.1	0.1
1475	1475	γ-Muurolene	---	tr	tr
1478	1481	(*E*)-β-Ionone	---	tr	tr
1481	1483	Germacrene D	0.2	0.3	0.2
1487	1487	β-Selinene	---	tr	---
1491	1491	Viridiflorene	---	tr	tr
1496	1497	Bicyclogermacrene	0.4	0.4	0.6
1504	1503	(*E*,*E*)-α-Farnesene	---	tr	tr
1513	1512	γ-Cadinene	---	tr	tr
1518	1518	δ-Cadinene	tr	0.1	0.1
1575	1574	Germacrene D-4-ol	0.1	0.1	0.2
1576	1577	Caryophyllene oxide	---	tr	tr
1585	1590	Globulol	---	tr	tr
1593	1594	Viridiflorol	---	tr	tr
1641	1643	τ-Cadinol	---	tr	tr
1641	1644	α-Muurolol (=δ-Cadinol)	---	---	tr
1643	1644	τ-Muurolol	---	tr	tr
1656	1655	α-Cadinol	0.1	0.1	0.1
		Monoterpene hydrocarbons	2.4	4.9	2.9
		Oxygenated monoterpenoids	1.0	0.1	0.1
		Sesquiterpene hydrocarbons	1.8	2.5	2.8
		Oxygenated sesquiterpenoids	0.2	0.2	0.3
		Benzenoid aromatics	94.1	91.3	92.9
		Others	0.5	1.0	0.9
		Total identified	100.0	100.0	100.0
		Compound	Enantiomeric Distribution (+):(-)		
			#1	#2	#3
		Sabinene	---	100:0	100:0
		1-Octen-3-ol	0:100	0:100	0:100
		Limonene	98.2:1.8	98.3:1.7	98.3:1.7
		β-Phellandrene	100:0	100:0	100:0
		Linalool	29.3:70.7	29.5:70.5	21.3:78.7
		*trans*-β-Elemene	---	60.4:39.6	57.9:42.1
		(*E*)-β-Caryophyllene	100:0	100:0	100:0
		Germacrene D	100:0	100:0	100:0
		δ-Cadinene	0:100	0:100	0:100

RI_calc_ = retention indices calculated with respect to a homologous series of *n*-alkanes on a ZB-5 column. RI_db_ = retention indices from the databases [29,30,31,32]. tr = trace (<0.05%).

**Table 3:** Essential oil composition of American wintergreen (*Gaultheria procumbens*) cultivated in southern Alabama.

RI_calc_	RI_db_	Compound	%
1033	1033	Benzyl alcohol	0.2
1051	1051	2,3,6-Trimethylhepta-1,5-diene	tr
1105	1104	Nonanal	tr
1160	1158	Menthone	0.1
1193	1192	Methyl salicylate	91.1
1215	1217	Coumaran	tr
1237	1237	Pulegone	0.6
1262	1263	(2*E*)-Decenal	tr
1271	1273	(*E*)-Cinnamaldehyde	tr
1289	1289	Thymol	0.3
1304	1304	(*E*)-Cinnamyl alcohol	0.8
1336	1339	Piperitenone	tr
1388	1390	*trans*-β-Elemene	tr
1417	1417	(*E*)-β-Caryophyllene	tr
1431	1432	*trans*-α-Bergamotene	tr
1445	1447	Geranyl acetone	0.1
1467	1463	Tuberolactone	5.3
1470	1471	Massoia lactone	1.3
1479	1480	Germacrene D	0.1
1502	1503	(*E*,*E*)-α-Farnesene	tr
1516	1518	δ-Cadinene	tr
1579	1577	Caryophyllene oxide	tr
1652	1655	α-Cadinol	tr
1679	1676	8-Hydroxyisobornyl isobutanoate	tr
1761	1762	Benzyl benzoate	tr
1863	1869	Benzyl salicylate	tr
		Monoterpene hydrocarbons	tr
		Oxygenated monoterpenoids	1.1
		Sesquiterpene hydrocarbons	0.1
		Oxygenated sesquiterpenoids	tr
		Benzenoid aromatics	92.1
		Others	6.6
		Total identified	99.9
		Enantiomeric Distribution	(+):(-)
		Menthone	0:100
		Pulegone	100:0
		(*E*)-β-Caryophyllene	100:0
		Germacrene D	100:0

RI_calc_ = retention indices calculated with respect to a homologous series of *n*-alkanes on a ZB-5 column. RI_db_ = retention indices from the databases [29,30,31,32]. tr = trace (<0.05%).

**Table 4:** Essential oil composition of early sunflower (*Heliopsis helianthoides*) cultivated in southern Alabama.

RI_calc_	RI_db_	Compound	Percent Composition ^a^	
			Average	St. dev.
801	801	Hexanal	1.0	0.2
815	817	4-Hydroxy-2-pentanone	0.9	0.3
829	831	Furfural	0.3	0.1
848	850	(2*E*)-Hexenal	1.0	0.2
879	876	5-Methyl-(3*Z*)-hexen-2-one	0.9	0.1
959	959	Benzaldehyde	0.3	0.0
987	989	2-Pentyl furan	0.4	0.1
1102	1104	Nonanal	1.0	0.0
1290	1289	Thymol	1.8	0.6
1298	1300	Carvacrol	1.5	0.1
1309	1309	4-Vinylguaicol	5.5	0.6
1335	1335	δ-Elemene	0.5	0.1
1383	1382	β-Bourbonene	2.7	0.1
1388	1390	*trans*-β-Elemene	0.5	0.0
1417	1422	β-Ylangene	0.9	0.1
1418	1417	(*E*)-β-Caryophyllene	2.7	0.0
1428	1430	β-Copaene	1.2	0.1
1443	1447	*iso*-Germacrene D	0.4	0.0
1454	1453	α-Humulene	0.5	0.0
1477	1481	(*E*)-β-Ionone	0.5	0.0
1480	1480	Germacrene D	42.4	1.2
1494	1497	Bicyclogermacrene	0.8	0.1
1497	1502	ε-Amorphene	0.3	0.0
1503	1499	(*Z*)-Dihydroapofarnesal	0.3	0.0
1509	---	Unidentified ^b^	1.1	0.0
1516	1518	δ-Cadinene	0.4	0.0
1523	1516	(*E*)-Dihydroapofarnesal	0.3	0.1
1524	1524	Dihydroactinidiolide	0.7	0.0
1557	1557	Germacrene B	0.7	0.0
1560	1560	(*E*)-Nerolidol	0.8	0.2
1566	1566	1,5-Epoxysalvial-4(14)-ene	0.3	0.0
1575	1576	Spathulenol	1.2	0.0
1580	1577	Caryophyllene oxide	1.5	0.0
1590	1593	Salvial-4(14)-en-1-one	0.6	0.0
1626	1629	*iso*-Spathulenol	2.7	0.0
1638	1644	*allo*-Aromadendrene epoxide	0.3	0.1
1653	1655	α-Cadinol	1.0	0.1
1683	1683	Germacra-4(15),5,10(14)-trien-1α-ol	1.4	0.2
1685	1685	Eudesma-4(15),7-dien-1β-ol	0.5	0.1
1689	---	Germacra-4(15),5,10(14)-trien-1β-ol	1.1	0.2
1735	1735	Mint sulfide	0.3	0.1
1763	---	Unidentified ^c^	1.4	0.0
1827	---	Unidentified ^d^	1.1	0.0
1840	1841	Phytone	1.6	0.0
2097	2098	γ-Stearolactone	2.1	0.3
2106	2102	Phytol	2.8	1.3
2273	---	Unidentified ^e^	2.3	0.8
		Monoterpene hydrocarbons	0.0	
		Oxygenated monoterpenoids	3.3	
		Sesquiterpene hydrocarbons	53.8	
		Oxygenated sesquiterpenoids	12.2	
		Diterpenoids	2.8	
		Benzenoid aromatics	5.8	
		Others	10.6	
		Total identified	88.6	
		Enantiomeric Distribution	(+):(-)	
		δ-Elemene	52.1:47.9	
		*trans*-β-Elemene	100:0	
		(*E*)-β-Caryophyllene	100:0	
		(*E*)-β-Ionone	100:0	
		Germacrene D	89.3:10.7	
		δ-Cadinene	0:100	
		(*E*)-Nerolidol	0:100	

RI_calc_ = retention indices calculated with respect to a homologous series of *n*-alkanes on a ZB-5 column. RI_db_ = retention indices from the databases [29,30,31,32]. ^a^ Average of three measurements. ^b^ MS(EI): 182(7%), 153(5%), 126(8%), 112(20%), 111(37%), 99(5%), 83(100%), 69(6%), 55(39%), 43(14%), 41(7%). ^c^ MS(EI): 236(2%), 193(20%), 180(10%), 175(15%), 165(18%), 147(35%), 137(26%), 123(36%), 121(24%), 109(25%), 105(26%), 95(50%), 93(38%), 81(100%), 79(28%), 69(31%), 55(38%), 43(30%), 41(41%). ^d^ MS(EI): 234(11%), 219(11%), 191(91%), 173(19%), 163(48%), 151(71%), 145(41%), 131(37%), 123(100%), 121(42%), 107(55%), 105(40%), 95(42%), 93(59%), 91(75%), 83(72%), 81(81%), 79(66%), 77(57%), 69(48%), 55(75%), 43(52%), 41(100%). ^e^ MS(EI): 161(4%), 147(15%), 133(10%), 119(14%), 105(23%), 95(47%), 93(51%), 91(45%), 80(51%), 79(100%), 67(66%), 55(32%), 43(10%), 41(44%).

**Table 5:** Chemical composition of *Liatris spicata* aerial parts essential oil.

RI_calc_	RI_db_	Compound	%	ED, (+):(−)
905	902	Santolinatriene	0.5	
934	932	α-Pinene	8.1	17.1:82.9
950	950	Camphene	0.6	100:0
973	971	Sabinene	0.9	
976	974	Hexanoic acid	0.9	
979	978	β-Pinene	3.1	52.3:47.7
990	989	Myrcene	13.7	
1030	1030	Limonene	2.1	33.4:66.6
1032	1031	β-Phellandrene	0.4	
1047	1045	(*E*)-β-Ocimene	0.5	
1100	1098	Perillene	0.3	
1101	1099	Linalool	0.5	52.9:47.1
1106	1104	Nonanal	0.3	
1115	1113	(*E*)-4,8-Dimethylnona-1,3,7-triene	0.3	
1146	1145	*trans*-Verbenol	0.7	
1163	1164	Pinocarvone	0.3	
1172	1170	Borneol	0.7	0:100
1181	1180	Terpinen-4-ol	0.2	0:100
1196	1196	Myrtenal	0.6	
1207	1205	Verbenone	0.7	0:100
1284	1282	Bornyl acetate	3.0	0:100
1309	1309	4-Vinylguaiacol	0.8	
1319	1322	(2*E*,4*E*)-Decadienal	0.4	
1323	1319	(3*E*)-Hexenyl tiglate	0.4	
1330	1329	Hexyl tiglate	0.3	
1384	1382	β-Bourbonene	0.4	
1388	1387	β-Cubebene	0.2	
1390	1390	*trans*-β-Elemene	1.1	
1416	1414	α-Cedrene	0.3	
1418	1422	β-Ylangene	0.4	
1419	1424	(*E*)-β-Caryophyllene	4.4	100:0
1424	1423	β-Cedrene	0.4	
1429	1427	γ-Elemene	0.7	
1433	1432	*trans*-α-Bergamotene	1.8	
1447	1447	Geranyl acetone	0.3	
1453	1452	(*E*)-β-Farnesene	0.3	
1455	1454	α-Humulene	1.9	
1475	1475	γ-Muurolene	0.4	
1478	1481	(*E*)-β-Ionone	0.2	
1481	1480	Germacrene D	23.7	100:0
1488	1489	β-Selinene	0.5	
1495	1497	Bicyclogermacrene	0.5	
1498	1497	α-Muurolene	0.3	
1518	1518	δ-Cadinene	0.8	0:100
1524	1524	Dihydroactinidiolide	0.4	
1558	1557	Germacrene B	0.6	
1560	1561	(*E*)-Nerolidol	0.5	0:100
1567	1566	1,5-Epoxysalvial-4(14)-ene	0.9	
1576	1576	Spathulenol	2.5	
1581	1577	Caryophyllene oxide	5.9	
1584	---	Unidentified ^a^	2.0	
1591	1593	Salvial-4(14)-en-1-one	1.2	
1608	1611	Humulene epoxide II	1.3	
1621	1620	*epi*-Cedrol	1.8	
1627	1629	*iso*-Spathulenol	1.6	
1654	1655	α-Cadinol	1.6	
		Monoterpene hydrocarbons	29.8	
		Oxygenated monoterpenoids	7.2	
		Sesquiterpene hydrocarbons	38.7	
		Oxygenated sesquiterpenoids	17.3	
		Benzenoid aromatics	0.8	
		Others	3.7	
		Total identified	97.4	

RI_calc_ = retention indices calculated with respect to a homologous series of *n*-alkanes on a ZB-5 column. RI_db_ = retention indices from the databases [29,30,31,32]. ED = enantiomeric distribution (dextrorotatory enantiomer/levorotatory enantiomer). ^a^ MS(EI): 220(19%), 202(16%), 178(18%), 177(33%), 164(29%), 159(71%), 149(37%), 135(45%), 131(34%), 121(44%), 117(48%), 107(75%), 105(66%), 93(100%), 91(97%), 81(50%), 79(69%), 77(50%), 67(47%), 55(65%), 43(56%), 41(78%).

**Table 6:** Chemical composition of *Pycnanthemum incanum* aerial parts essential oil.

RI_calc_	RI_db_	Compound	%	ED, (+):(−)	[20]
933	933	α-Pinene	tr		29:71
972	971	Sabinene	0.5	0:100	
978	978	β-Pinene	0.1	0:100	
979	978	1-Octen-3-ol	3.1	0:100	0:100
989	989	Myrcene	0.3		
1018	1017	α-Terpinene	0.1	100:0	
1025	1024	*p*-Cymene	0.8		
1030	1030	Limonene	1.2	0:100	
1032	1030	1,8-Cineole	30.7		
1036	1034	(*Z*)-β-Ocimene	0.1		
1046	1046	(*E*)-β-Ocimene	0.2		
1058	1058	γ-Terpinene	0.6		
1070	1069	*cis*-Sabinene hydrate	5.6	7.2:92.8	
1086	1086	Terpinolene	0.2		
1100	1099	Linalool	0.3		95:5
1101	1099	*trans*-Sabinene hydrate	3.0	36.5:63.5	
1125	1124	*cis-p*-Menth-2-en-1-ol	0.2		
1142	1139	*trans-p*-Menth-2-en-1-ol	0.1		
1156	1156	Menthone	0.2		0:100
1164	1165	Isomenthone	1.0	100:0	100:0
1170	1170	δ-Terpineol	2.6		
1171	1170	Borneol	8.2	0:100	
1180	1180	Terpinen-4-ol	1.8	33.1:66.9	
1195	1195	α-Terpineol	16.9	29.2:70.8	15:85
1237	1237	Pulegone	1.8	100:0	100:0
1283	1282	Bornyl acetate	0.2	0:100	
1289	1289	Thymol	0.3		
1331	1331	Bicycloelemene	0.1		
1335	1335	δ-Elemene	0.6	0:100	
1375	1375	α-Copaene	0.2	100:0	
1383	1382	β-Bourbonene	0.2		
1389	1390	*trans*-β-Elemene	0.3	47.0:53.0	
1418	1417	(*E*)-β-Caryophyllene	11.0	100:0	
1429	1430	β-Copaene	0.1		
1454	1453	α-Humulene	0.5		
1479	1480	Germacrene D	5.0	90.9:9.1	
1494	1497	Bicyclogermacrene	0.3		
1503	1504	(*E*,*E*)-α-Farnesene	0.2		
1517	1520	δ-Cadinene	0.3	0:100	
1557	1557	Germacrene B	0.1		
1559	1561	(*E*)-Nerolidol	0.1	0:100	
1575	1576	Germacrene D-4-ol	0.1		
1580	1577	Caryophyllene oxide	0.3		
1637	1639	*cis*-Guaia-3,9-dien-11-ol	0.3		
		Monoterpene hydrocarbons	4.3		
		Oxygenated monoterpenoids	72.9		
		Sesquiterpene hydrocarbons	18.8		
		Oxygenated sesquiterpenoids	0.9		
		Others	3.1		
		Total identified	100.0		

RI_calc_ = retention indices calculated with respect to a homologous series of *n*-alkanes on a ZB-5 column. RI_db_ = retention indices from the databases [29,30,31,32]. ED = enantiomeric distribution (dextrorotatory enantiomer/levorotatory enantiomer). tr = trace (<0.05%).

**Table 7:** Essential oil composition of *Smallanthus uvedalia* from South Carolina.

RI_calc_	RI_db_	Compound	%	ED, (+):(−)
801	801	Hexanal	0.1	
844	842	Isovaleric acid	1.7	
849	850	(2*E*)-Hexenal	0.6	
851	853	(3*Z*)- Hexenol	0.1	
922	923	Tricyclene	tr	
925	925	α-Thujene	0.1	19.1:80.9
935	933	α-Pinene	23.9	6.7:93.3
948	950	Camphene	0.4	100:0
952	953	Thuja-2,4(10)-diene	tr	
960	960	Benzaldehyde	0.1	
972	972	Sabinene	3.4	18.3:81.7
977	978	β-Pinene	0.9	30.7:69.3
988	989	Myrcene	1.0	
1016	1017	α-Terpinene	0.1	
1024	1025	*p*-Cymene	tr	
1030	1030	Limonene	6.1	95.2:4.8
1031	1031	β-Phellandrene	0.2	100:0
1032	1032	1,8-Cineole	tr	
1033	1033	Benzyl alcohol	tr	
1034	1034	(*Z*)-β-Ocimene	tr	
1042	1043	Benzene acetaldehyde	0.1	
1045	1045	(*E*)-β-Ocimene	0.4	
1057	1057	γ-Terpinene	0.2	
1069	1069	*cis*-Sabinene hydrate	0.1	0:100
1084	1086	Terpinolene	0.1	
1089	1091	*p*-Cymenene	tr	
1091	1091	Rosefuran	0.1	
1101	1098	Perillene	14.5	
1102	1101	*trans*-Sabinene hydrate	0.1	0:100
1105	1104	Nonanal	tr	
1111	1111	Phenethyl alcohol	0.1	
1112	1113	(*E*)-1,4-Dimethylnona-1,3,7-triene	0.2	
1121	1121	*trans-p*-Mentha2,8-dien-1-ol	tr	
1124	1124	*cis-p*-Menth-2-en-1-ol	tr	
1126	1125	α-Campholenal	0.1	
1139	1140	*trans*-Pinocarveol	0.1	
1141	1141	*cis*-Verbenol	0.1	
1145	1145	*trans*-Verbenol	0.4	
1161	1164	Pinocarvone	0.1	
1170	1171	*p*-Mentha-1,5-dien-8-ol	tr	
1179	1180	Terpinen-4-ol	0.4	28.8:71.2
1186	1186	*p*-Cymen-8-ol	tr	
1194	1195	α-Terpineol	0.2	34.5:65.5
1205	1205	Verbenone	0.1	89.3:10.7
1217	1218	*trans*-Carveol	0.1	
1288	1289	Thymol	0.1	
1331	1334	Bicycloelemene	0.2	
1335	1336	δ-Elemene	0.1	
1351	1356	Eugenol	0.1	
1359	1361	Neryl acetate	0.1	
1375	1375	α-Copaene	0.1	100:0
1378	1379	(*E*)-β-Damascenone	0.1	
1382	1383	*cis*-β-Elemene	0.1	
1383	1382	β-Bourbonene	0.5	
1386	1385	α-Bourbonene	tr	
1387	1387	β-Cubebene	tr	
1389	1390	*trans*-β-Elemene	1.0	17.1:82.9
1403	1405	(*Z*)-β-Caryophyllene	tr	
1415	1416	*cis*-α-Bergamotene	tr	
1423	1424	(*E*)-β-Caryophyllene	16.9	100:0
1429	1427	γ-Elemene	1.3	
1433	1432	*trans*-α-Bergamotene	0.1	
1438	1438	Aromadendrene	0.1	
1441	1444	Guaia-6,9-diene	0.3	
1443	1447	*iso*-Germacrene D	0.1	
1448	1447	Geranyl acetone	0.1	
1450	1453	ε-Muurolene	0.1	
1455	1454	α-Humulene	1.1	
1467	1464	9-*epi*-(*E*)-Caryophyllene	tr	
1476	1478	γ-Muurolene	0.2	
1483	1480	Germacrene D	12.2	96.4:3.6
1489	1489	β-Selinene	tr	
1490	1493	Phenethyl isovalerate	0.1	
1492	1490	γ-Amorphene	tr	
1495	1497	Bicyclogermacrene	1.9	
1498	1497	α-Muurolene	0.1	
1502	1504	Epizonarene	0.1	
1504	1505	(*E*,*E*)-α-Farnesene	0.2	
1507	1508	β-Bisabolene	0.1	
1512	1512	γ-Cadinene	0.1	
1517	1518	δ-Cadinene	0.3	0:100
1526	1528	(*E*)-γ-Bisabolene	0.2	
1536	1538	α-Cadinene	0.1	
1536	1531	(*Z*)-Nerolidol	0.1	
1548	1549	α-Elemol	0.1	
1558	1557	Germacrene B	2.1	
1561	1560	(*E*)-Nerolidol	0.3	34.1:65.9
1576	1576	Spathulenol	0.9	
1581	1577	Caryophyllene oxide	1.3	
1631	1630	Caryophylla-4(12),8(13)-dien-5α-ol	0.1	
1636	1636	Caryophylla-4(12),8(13)-dien-5β-ol	0.2	
1641	1640	τ-Cadinol	0.1	
1643	1644	τ-Muurolol	0.1	
1654	1655	α-Cadinol	0.4	
1657	1660	Selin-11-en-4α-ol (=Kongol)	0.1	
1683	1683	Germacra-4(15),5,10(14)-trien-1α-ol	0.1	
1715	1715	Pentadecanal	0.1	
1841	1841	Phytone	0.2	
2020	2022	(*E*,*E*)-Geranyl linalool	0.1	
		Monoterpene hydrocarbons	36.7	
		Oxygenated monoterpenoids	16.5	
		Sesquiterpene hydrocarbons	39.2	
		Oxygenated sesquiterpenoids	3.8	
		Diterpenoids	0.1	
		Benzenoid aromatics	0.4	
		Others	3.2	
		Total identified	99.9	

RI_calc_ = retention indices calculated with respect to a homologous series of *n*-alkanes on a ZB-5 column. RI_db_ = retention indices from the databases [29,30,31,32]. ED = enantiomeric distribution (dextrorotatory enantiomer/levorotatory enantiomer). tr = trace (<0.05%).

**Table 8:** Essential oil compositions of three samples of wild-growing *Verbena hastata*.

RI_calc_	RI_db_	Compound	Percent Composition		
			#1	#2	#3
801	801	Hexanal	0.4	0.2	---
850	853	(3*Z*)-Hexenol	0.8	0.6	---
902	905	Heptanal	1.1	0.3	---
960	960	Benzaldehyde	0.3	0.4	---
974	973	1-Octen-3-one	0.7	0.3	---
977	978	1-Octen-3-ol	29.1	22.5	2.4
983	984	3-Octanone	0.2	0.2	---
988	989	2-Pentylfuran	0.3	---	---
991	995	6-Methyl-5-hepten-2-ol	0.3	0.2	---
995	996	3-Octanol	0.3	0.2	---
1002	1005	Octanal	0.3	0.1	---
1022	1025	*p*-Cymene	---	0.5	---
1027	1030	Limonene	---	0.1	---
1032	1033	Benzyl alcohol	0.3	0.9	0.3
1068	1069	*cis*-Linalool oxide (furanoid)	---	0.7	---
1068	1076	1-Octanol	1.0	---	0.6
1083	1082	Terpinolene	---	0.1	---
1084	1086	*trans*-Linalool oxide (furanoid)	---	0.3	0.1
1098	1101	Linalool	1.5	1.9	0.7
1103	1104	Nonanal	11.1	1.8	2.0
1110	1111	Phenyl ethyl alcohol	1.7	2.3	1.2
1184	1186	*p*-Cymen-8-ol	---	0.2	0.1
1189	1190	Methyl salicylate	0.4	1.0	0.4
1193	1195	α-Terpineol	---	0.3	0.3
1196	1197	Methyl chavicol (=Estragole)	1.0	0.3	2.8
1204	1206	Decanal	---	0.2	0.2
1214	1217	Coumaran	---	0.2	0.5
1216	1219	β-Cyclocitral	---	0.5	0.2
1263	1263	(2*E*)-Decenal	0.3	0.1	0.2
1267	1272	Nonanoic acid	0.8	1.0	2.6
1274	1271	Decanol	0.3	---	0.6
1290	1293	Thymol	0.3	0.2	1.1
1298	1300	Carvacrol	0.3	0.1	0.4
1309	1309	4-Vinylguaiacol	---	0.2	0.6
1351	1356	Eugenol	---	0.3	0.3
1378	1380	(*E*)-β-Damascenone	0.5	0.8	0.9
1408	1410	Italicene	---	---	0.4
1447	1447	Geranyl acetone	0.3	0.6	0.6
1461	1461	4,6,8,10-Tetramethyltridecane	0.8	0.3	0.7
1477	1481	(*E*)-β-Ionone	0.6	0.8	0.8
1480	1480	5,6-Epoxy-β-ionone	0.3	0.7	0.4
1524	1524	Dihydroactinidiolide	0.5	0.9	1.2
1560	1560	(*E*)-Nerolidol	---	---	0.2
1596	1596	Fokienol	0.3	1.0	1.1
1600	1600	Hexadecane	0.3	---	0.2
1628	1627	Eremoligenol	0.3	---	0.2
1630	1632	γ-Eudesmol	1.0	---	1.1
1638	1640	Hinesol	0.2	---	---
1653	2656	β-Eudesmol	2.1	0.9	1.8
1690	1694	Germacrone	---	0.4	---
1714	1715	Pentadecanal	0.3	---	0.4
1758	1758	Myristic acid	---	0.8	---
1840	1841	Phytone	4.4	3.1	7.3
1866	1869	Benzyl salicylate	1.1	2.0	1.8
1889	1891	Hexadecatrienal	---	---	0.3
1904	1902	(*E*,*E*)-6,10,14-Trimethyl-5,9,13-pentadecatrien-2-one	---	---	0.3
1921	1921	Methyl palmitate	---	---	0.3
1959	1958	Palmitic acid	8.5	21.6	15.3
2019	2018	Octadecanal	0.2	---	0.4
2082	2081	1-Octadecanol	9.6	2.8	14.0
2094	2098	Methyl linolenate	---	---	0.3
2106	2106	Phytol	5.2	7.1	12.6
2128	2128	Linoleic acid	---	0.8	0.6
2132	2134	α-Linolenic acid	1.1	4.4	3.2
2300	2300	Tricosane	0.7	0.5	1.0
2500	2500	Pentacosane	0.4	0.4	0.6
2700	2700	Heptacosane	0.4	0.3	0.5
		Monoterpenoids	2.0	4.9	2.9
		Sesquiterpenoids	3.9	2.3	4.9
		Diterpenoids	5.2	7.1	12.6
		Benzenoids	4.9	7.5	7.9
		Others	75.7	66.3	58.1
		Total identified	91.7	88.2	86.5
		Enantiomeric distribution	(+):(−)		
		1-Octen-3-ol	0:100	0:100	0:100
		Linalool	22.9:77.1	22.2:77.8	22.7:77.3
		α-Terpineol	49.6:50.4	49.8:50.2	49.9:50.1
		(*E*)-β-Ionone	100:0	100:0	100:0
		(*E*)-Nerolidol	0:100	0:100	0:100

RI_calc_ = retention indices calculated with respect to a homologous series of *n*-alkanes on a ZB-5 column. RI_db_ = retention indices from the databases [29,30,31,32].

## Data Availability

All data are presented in this report.

## Supplementary Materials

The following are available online at https://www.mdpi.com/article/10.3390/plants10061061/s1, Table S1: Enantiomeric distribution of terpenoids in Asteraceae species, Figure S1: Chiral gas chromatogram of *Agastache foeniculum* essential oil, Figure S2: Chiral gas chromatogram of *Gaultheria procumbens* essential oil, Figure S3: Chiral gas chromatogram of *Helianthus helianthoides* essential oil, Figure S4: Chiral gas chromatogram of *Liatris spicata* essential oil, Figure S5: Chiral gas chromatogram of *Pycnanthemum incanum* essential oil, Figure S6: Chiral gas chromatogram of *Smallanthus uvedalia* essential oil, Figure S7: Chiral gas chromatogram of *Verbena hastata* essential oil.
