# Supplementary material for: The Volatile Phytochemistry of Seven Native American Aromatic Medicinal Plants
Source: Plants (Basel). 2021 May 25;10(6):1061. doi: 10.3390/plants10061061 (PMC8229852; doi:10.3390/plants10061061)
Supplement: Supplementary file 1 [file plants-10-01061-s001.zip › Supplementary Table S1.pdf]

Supplementary Table S1. Enantiomeric distribution of terpenoids in Asteraceae species.

Compound	*Heliopsis helianthoides*	*Liatris spicata*	*Smallanthus uvedalia*	*Achillea ligustica*	*Erechtites hieracifolia*	*Solidago canadensis*	*Coreopsis triloba*	*Baccharis tridentata* Vahl	
								male	female
		this work		[1]	[2]	[3]	[4]	[5]	
α-Thujene	---	---	19.1:80.9	---	---	---	---	3:97	5:95
α-Pinene	---	17.1:82.9	6.7:93.3	59:41	100:0	74:26	18.4:81.6	15.3:84.7	16:84
Camphene	---	100:0	100:0	78:22	---	---	---	4:96	3:97
Sabinene	---	---	8.5:91.5	---	---	---	---	23:77	24:76
β-Pinene	---	52.3:47.7	30.7:69.3	2:98	10.3:89.7	36:64	100:0	73:27	73:27
Limonene	---	33.4:66.6	95.2:4.8	---	0:100	98:2	2.5:97.5	66.3:33.7	66.0:34.0
β-Phellandrene	---	---	100:0	---	---	---	---	---	---
cis-Sabinene hydrate	---	---	0:100	---	---	---	---	---	---
Linalool	---	52.9:47.1	---	---	75.9:24.1	---	---	---	---
trans-Sabinene hydrate	---	---	0:100	---	---	---	---	---	---
Borneol	---	0:100	---	27:73	---	---	---	---	---
Terpinen-4-ol	---	0:100	28.8:71.2	56:44	---	---	---	---	---
α-Terpineol	---	---	34.5:65.5	---	---	---	---	---	---
Verbenone	---	0:100	91.1:8.9	---	---	---	---	---	---
Bornyl acetate	---	0:100	---	---	---	0:100	---	---	---
α-Copaene	---	---	100:0	---	---	---	---	---	---
δ-Elemene	50.0:50.0	---	---	---	---	---	---	---	---
trans-β-Elemene	100:0	---	17.1:82.9	---	---	---	---	---	---
(*E*)-β-Caryophyllene	100:0	100:0	100:0	---	---	---	---	---	---
(*E*)-β-Ionone	100:0	---	---	---	---	---	---	---	---
Germacrene D	89.3:10.7	100:0	96.4:3.6	---	---	71:29	0:100	---	---
δ-Cadinene	0:100	0:100	0:100	---	---	---	---	---	---
(*E*)-Nerolidol	0:100	0:100	34.1:65.9	---	---	---	---	---	---

1. Filippi, J.-J.; Lanfranchi, D.-A.; Prado, S.; Baldovini, N.; Meierhenrich, U.J. Composition, enantiomeric distribution, and antibacterial activity of the essential oil of Achillea ligustica All. from Corsica. *J. Agric. Food Chem.* **2006**, *54*, 6308–6313,

doi:10.1021/jf060752y.
2. Lorenzo, D.; Saavedra, G.; Loayza, I.; Dellacassa, E. Composition of the essential oil of Erechtites hieracifolia from Bolivia. *Flavour Fragr. J.* **2001**, *16*, 353–355, doi:10.1002/ffj.1010.
3. Chanotiya, C.S.; Yadav, A. Natural variability in enantiomeric composition of bioactive chiral terpenoids in the essential oil of Solidago canadensis L. from Uttarakhand, India. *Nat. Prod. Commun.* **2008**, *3*, 263–266.
4. Espinosa, S.; Bec, N.; Larroque, C.; Ramírez, J.; Sgorbini, B.; Bicchi, C.; Gilardoni, G. Chemical, enantioselective, and sensory analysis of a cholinesterase inhibitor essential oil from Coreopsis triloba S.F. Blake (Asteraceae). *Plants* **2019**, *8*, 448, doi:10.3390/plants8110448.
5. Minteguiaga, M.; Fariña, L.; Cassel, E.; Fiedler, S.; Catalán, C.A.N.; Dellacassa, E. Chemical compositions of essential oil from the aerial parts of male and female plants of Baccharis tridentata Vahl. (Asteraceae). *J. Essent. Oil Res.* **2021**, in press, doi:10.1080/10412905.2020.1829720.
