# Supplementary figures and images for: The Volatile Phytochemistry of Seven Native American Aromatic Medicinal Plants
Source: Plants (Basel). 2021 May 25;10(6):1061. doi: 10.3390/plants10061061 (PMC8229852; doi:10.3390/plants10061061)

**Supplementary Figure S1.** Chiral gas chromatogram of *Agastache foeniculum* essential oil.

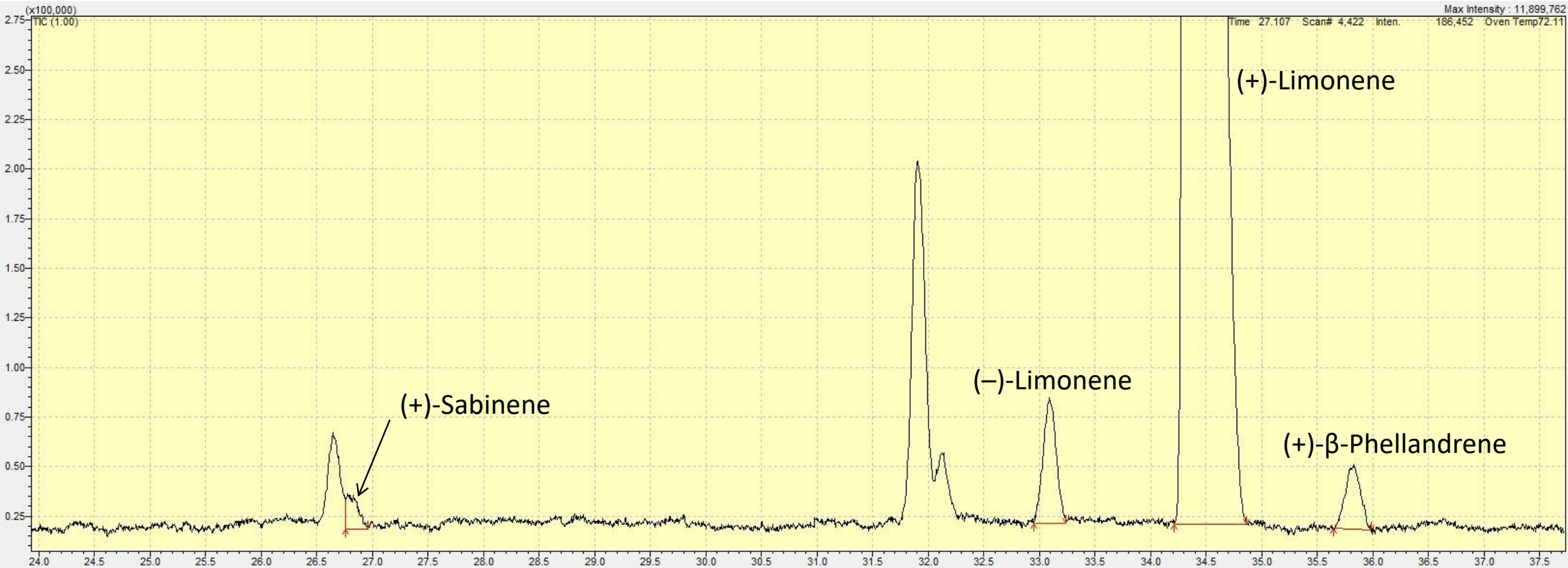

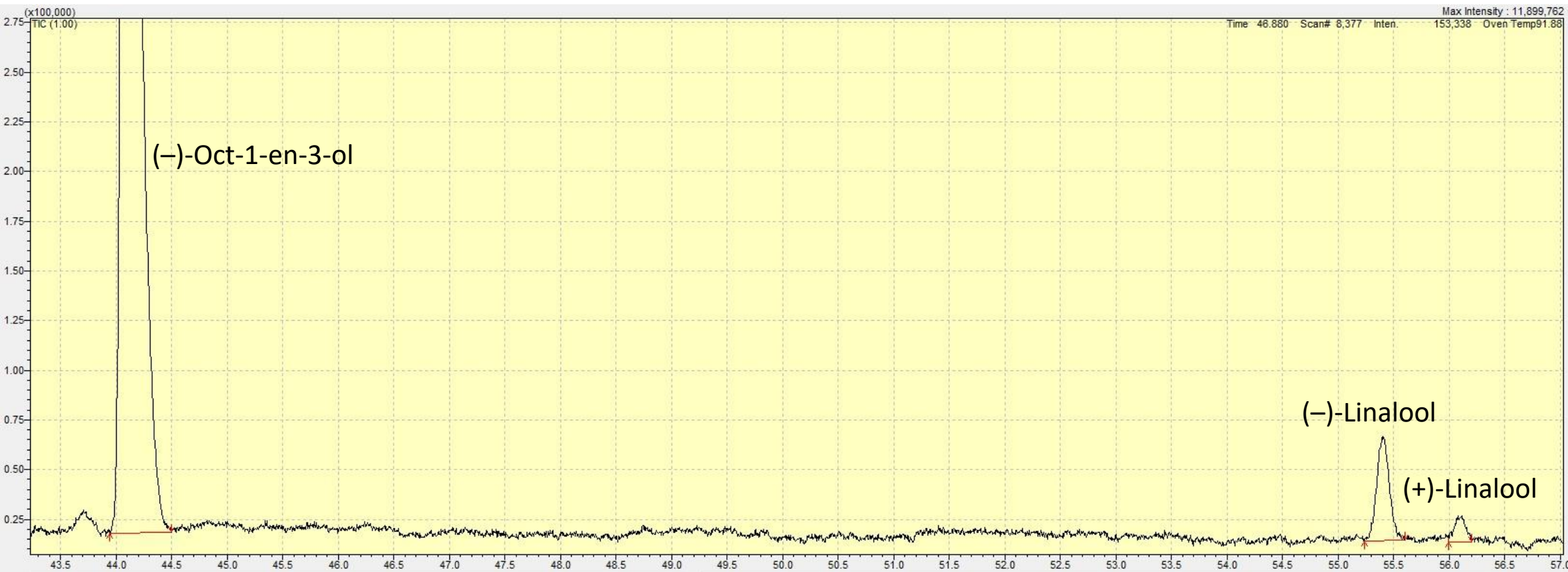

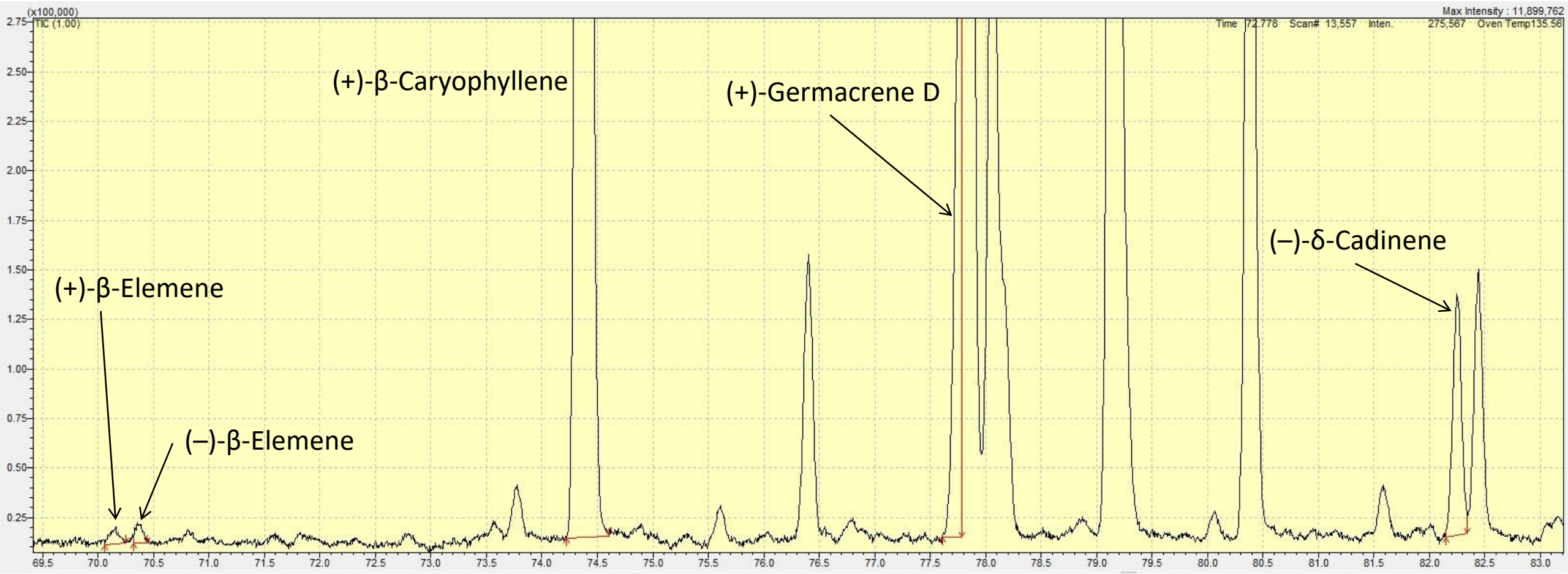

Supplement: Supplementary file 1 [file plants-10-01061-s001.zip › Figure_S1_A_foeniculum_chiral_GC-MS.pdf]

**Supplementary Figure S2.** Chiral gas chromatogram of *Gaultheria produmbens* essential oil.

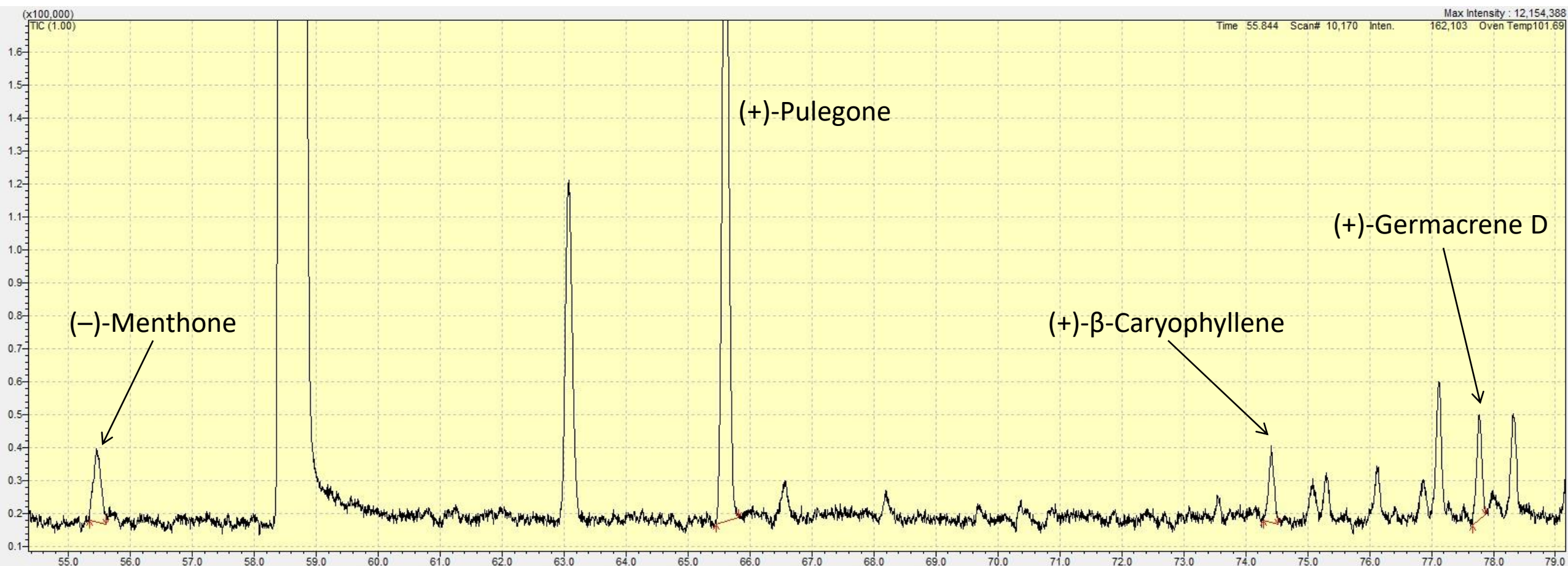

Supplement: Supplementary file 1 [file plants-10-01061-s001.zip › Figure_S2_G_procumbens_chiral_GC-MS.pdf]

**Supplementary Figure S3.** Chiral gas chromatogram of *Helianthus helianthoides* essential oil.

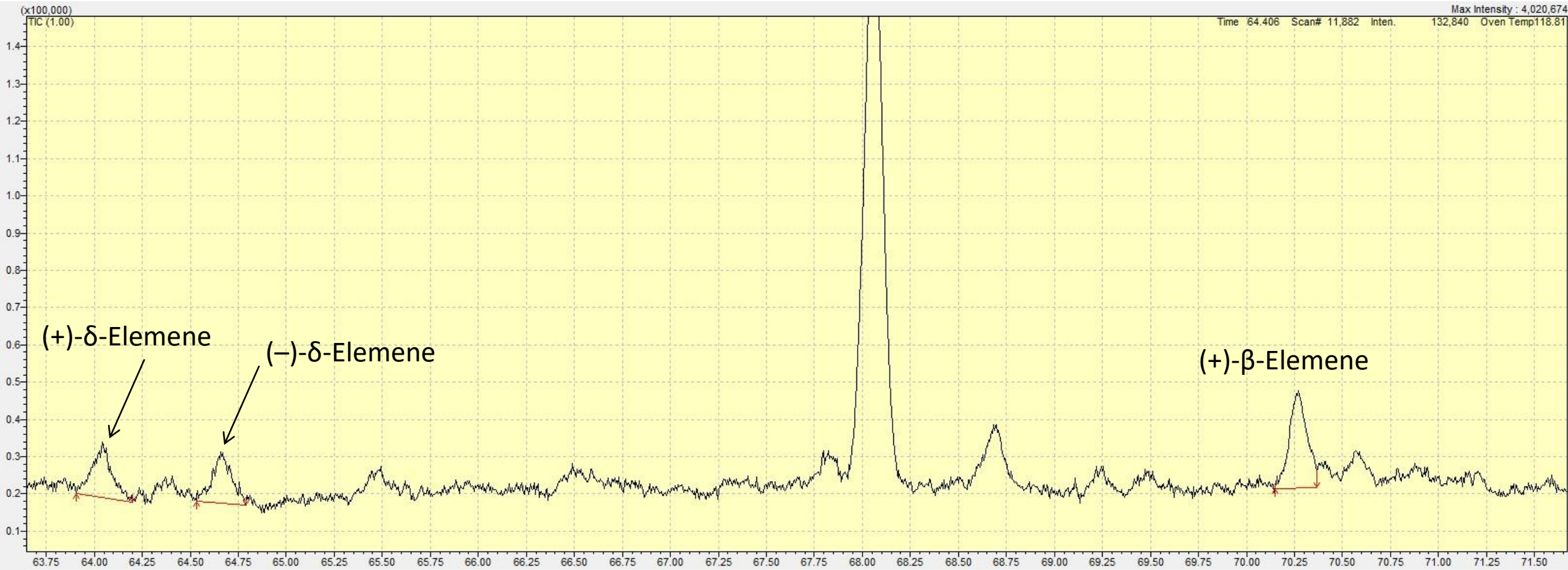

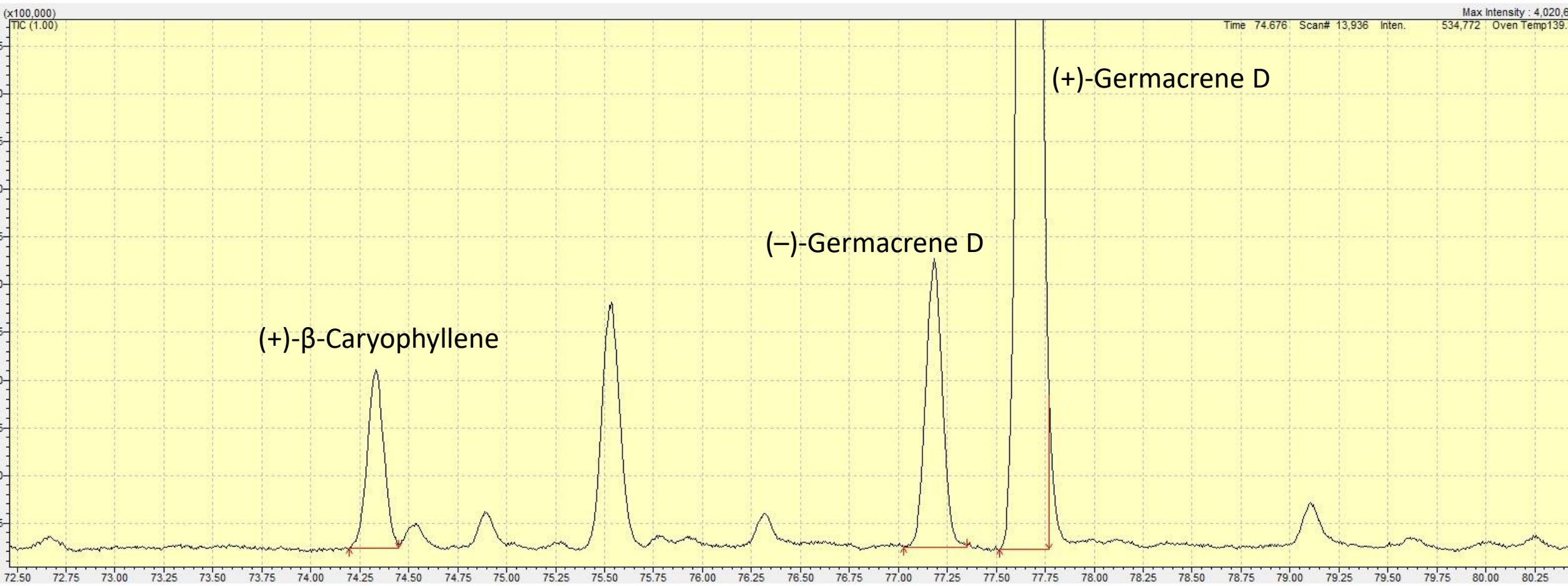

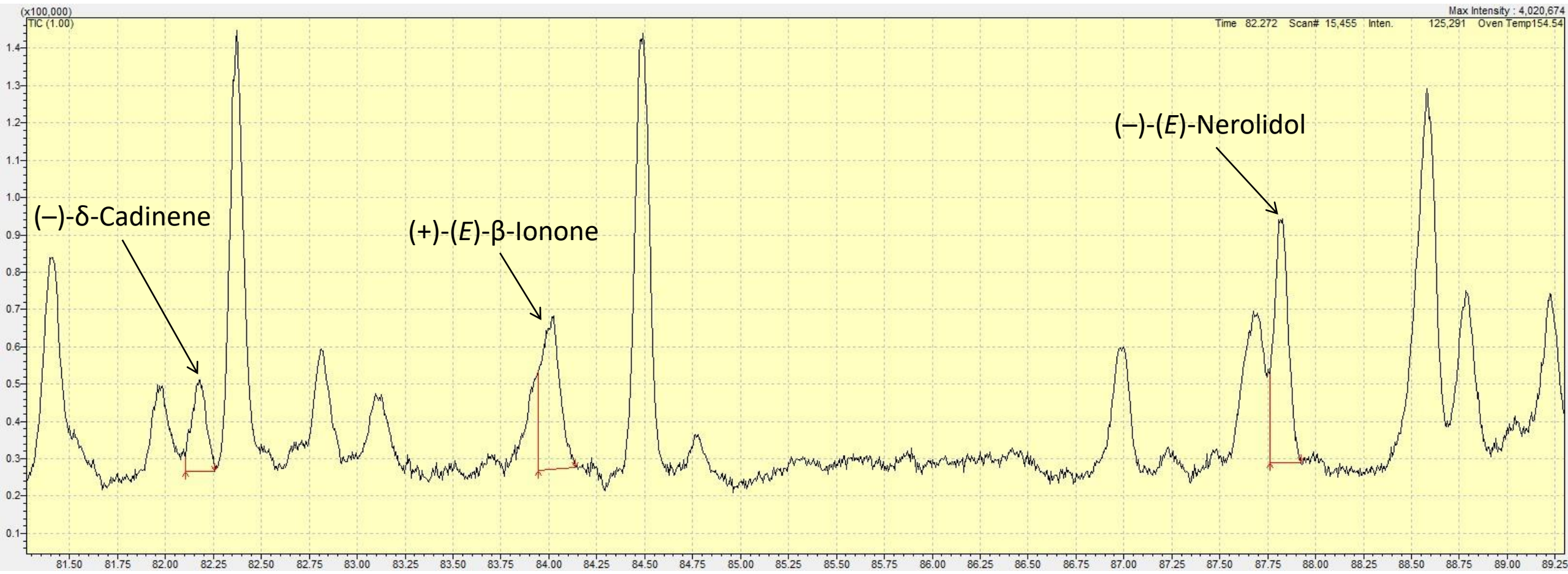

Supplement: Supplementary file 1 [file plants-10-01061-s001.zip › Figure_S3_H_helianthoides_chiral_GC-MS.pdf]

**Supplementary Figure S4.** Chiral gas chromatogram of *Liatris spicata* essential oil.

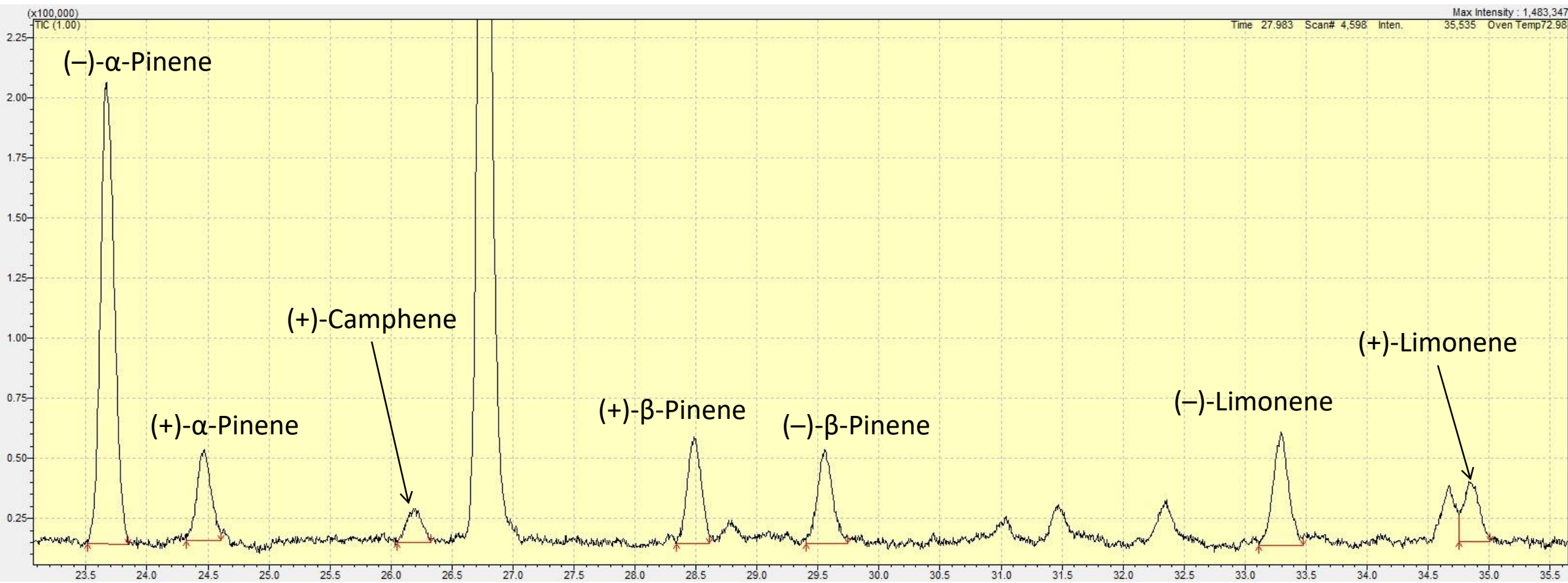

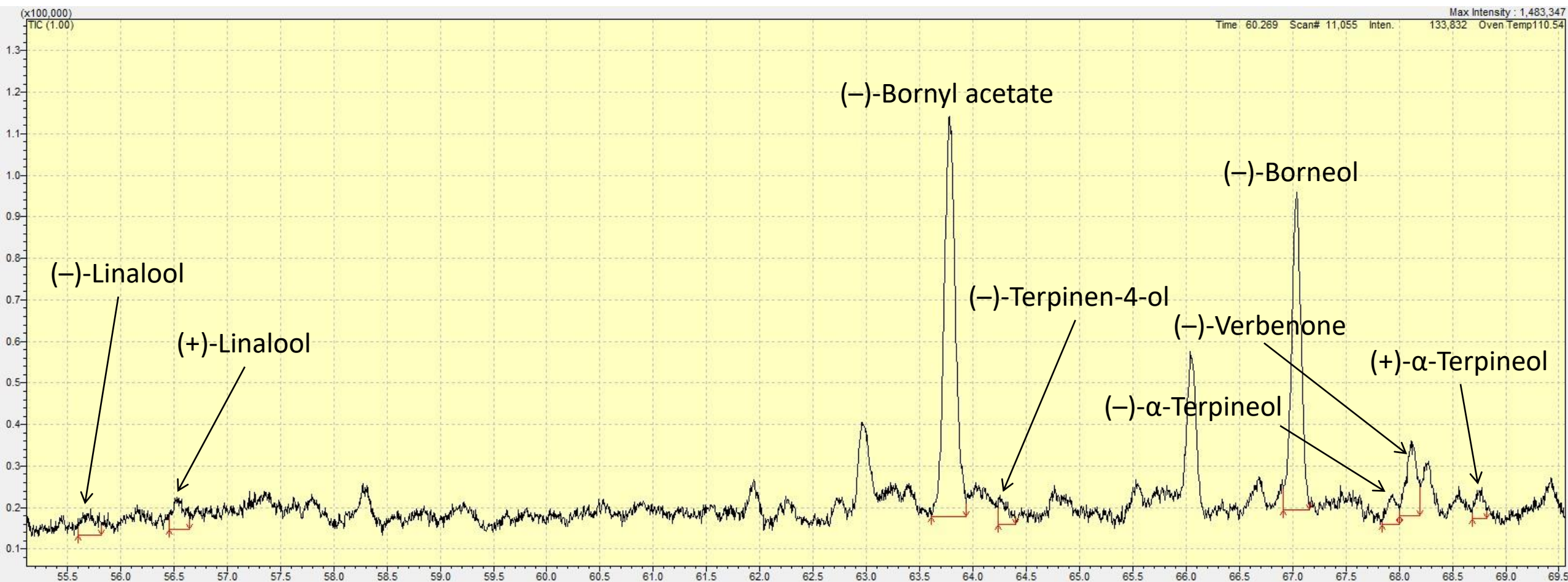

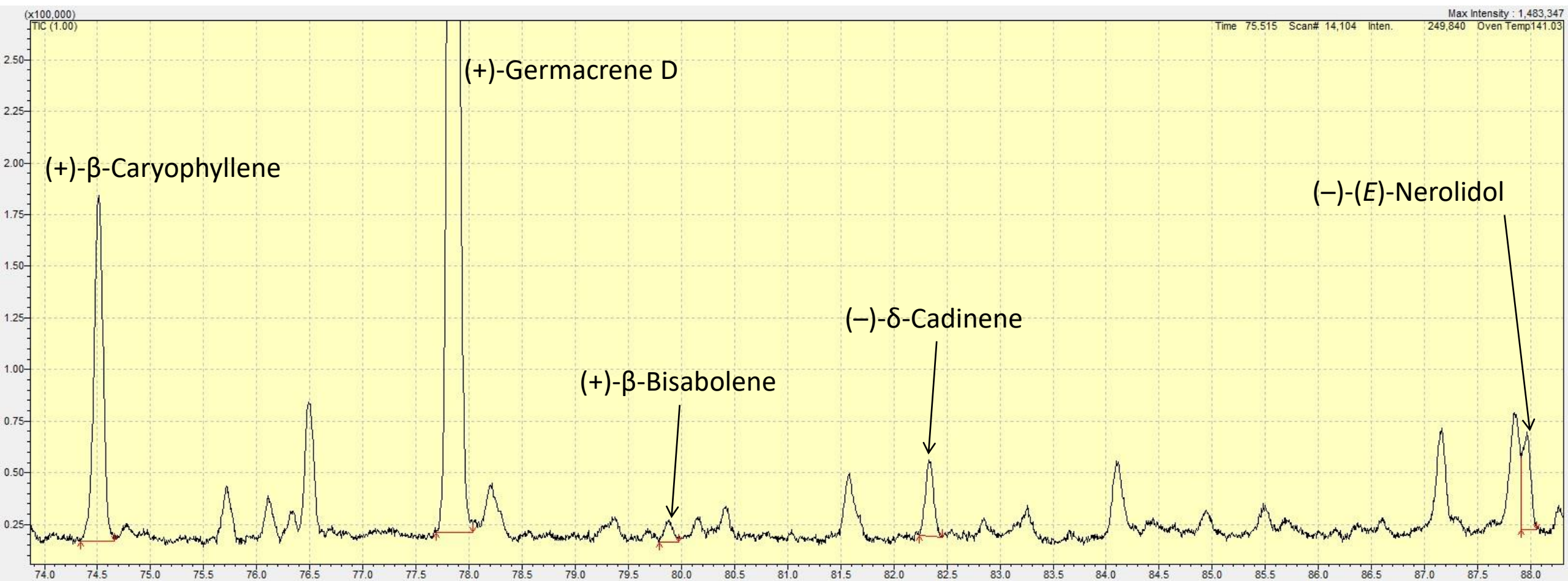

Supplement: Supplementary file 1 [file plants-10-01061-s001.zip › Figure_S4_L_spicata_chiral_GC-MS.pdf]

**Supplementary Figure S5.** Chiral gas chromatogram of *Pycnanthemum incanum* essential oil.

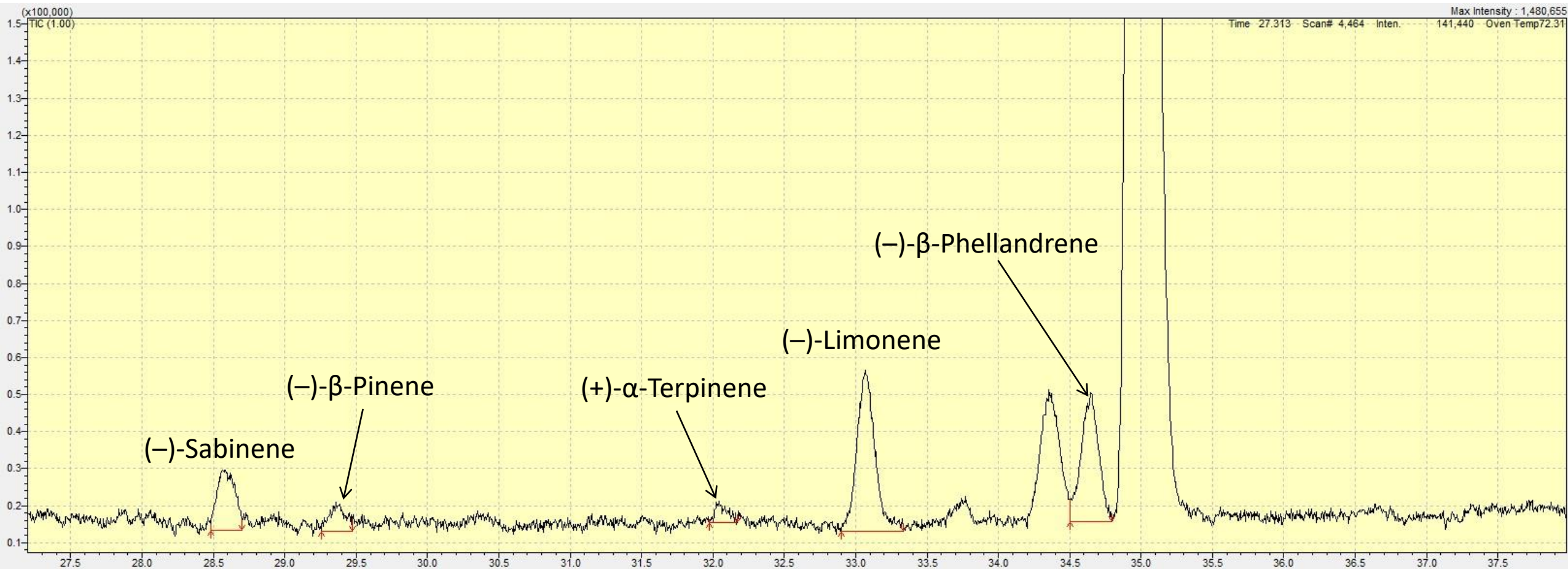

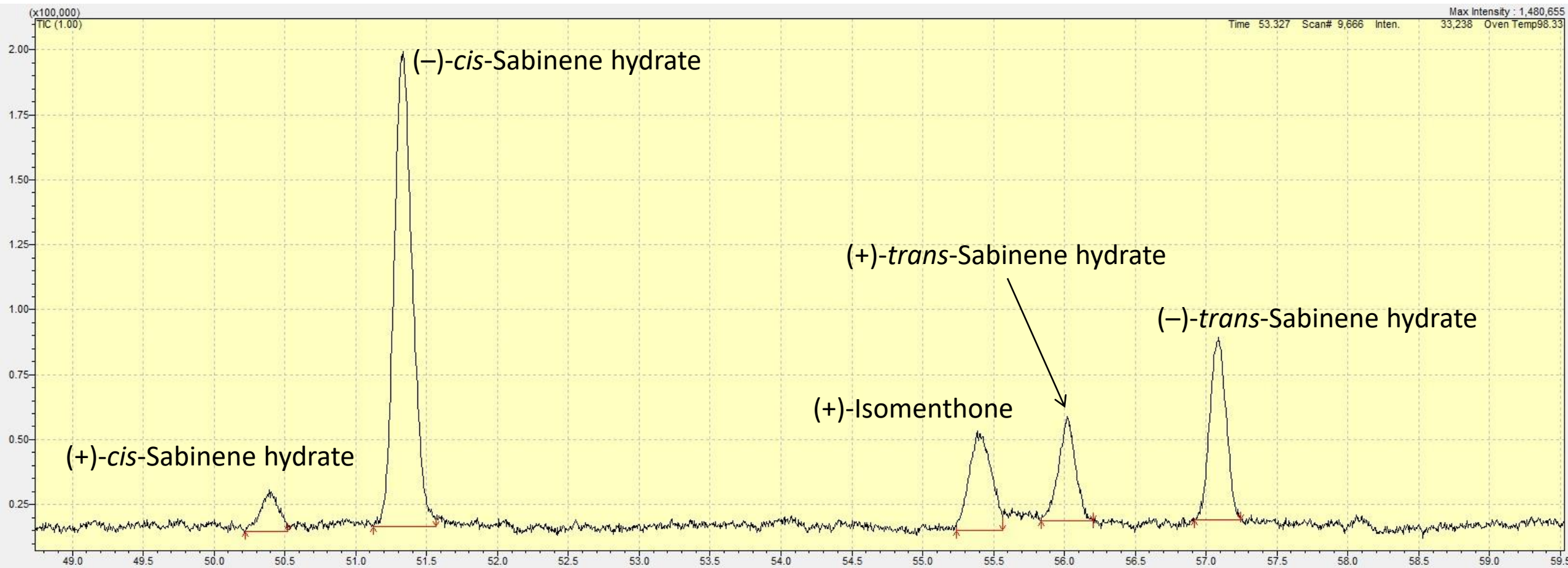

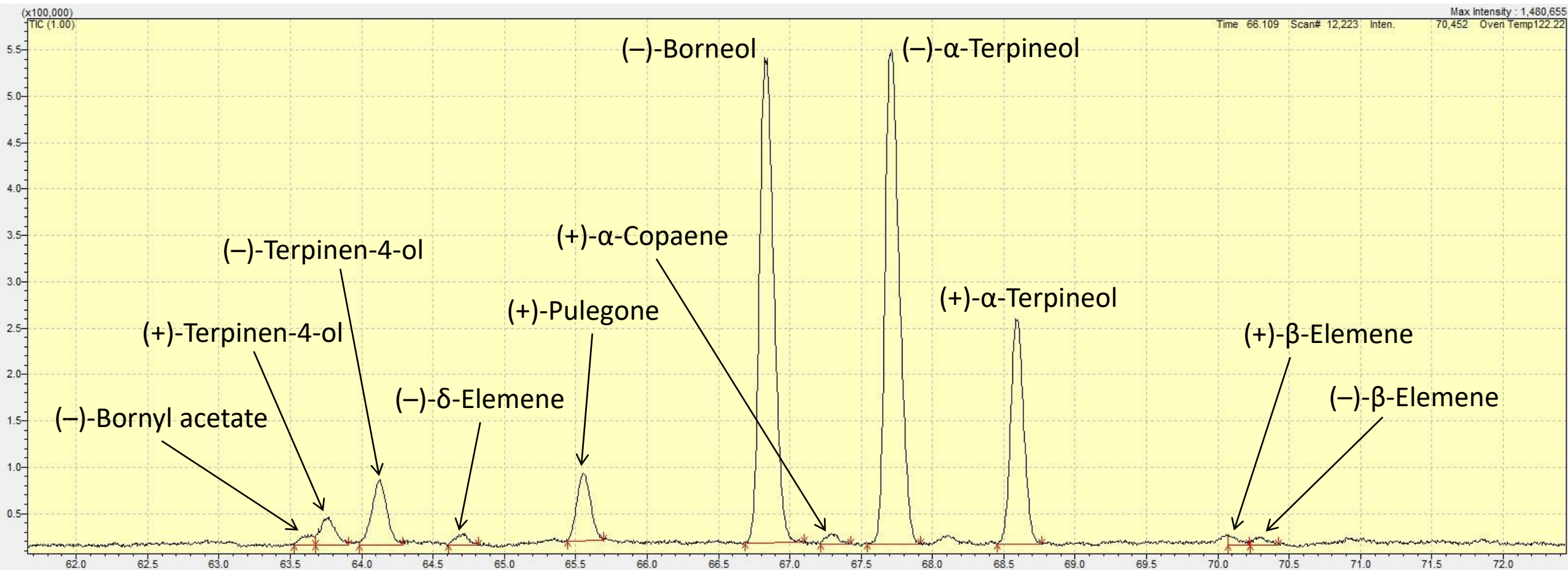

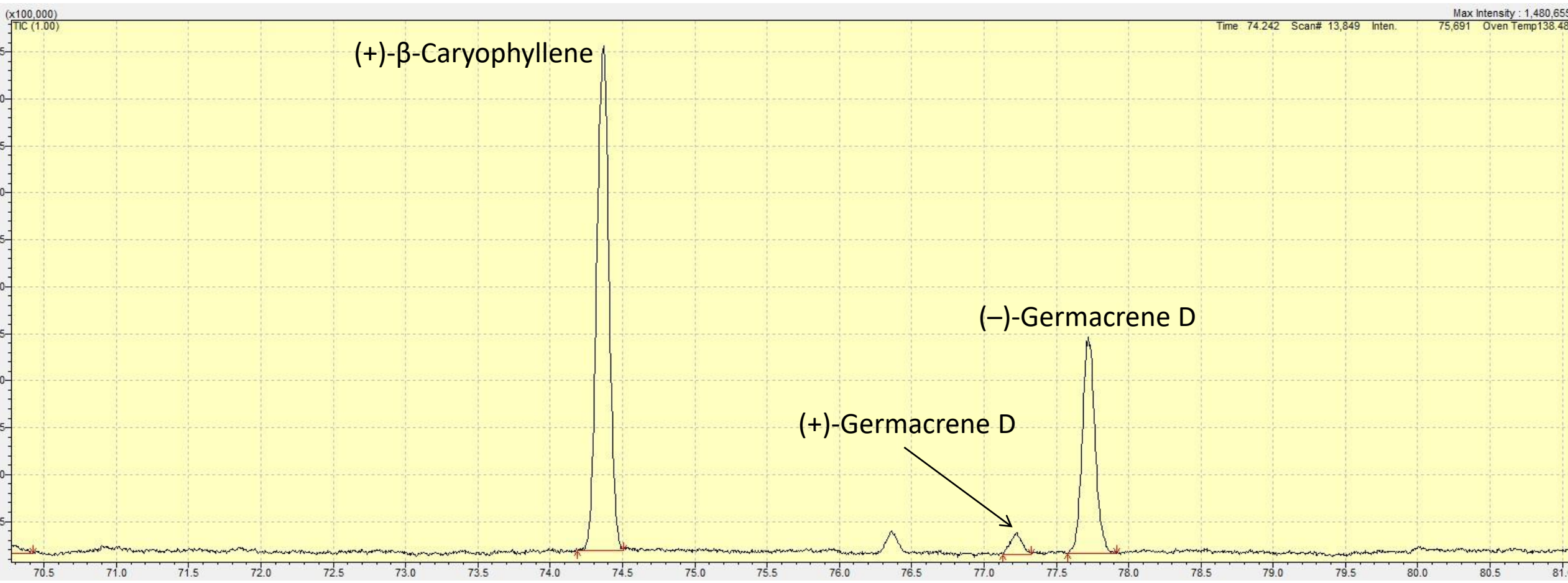

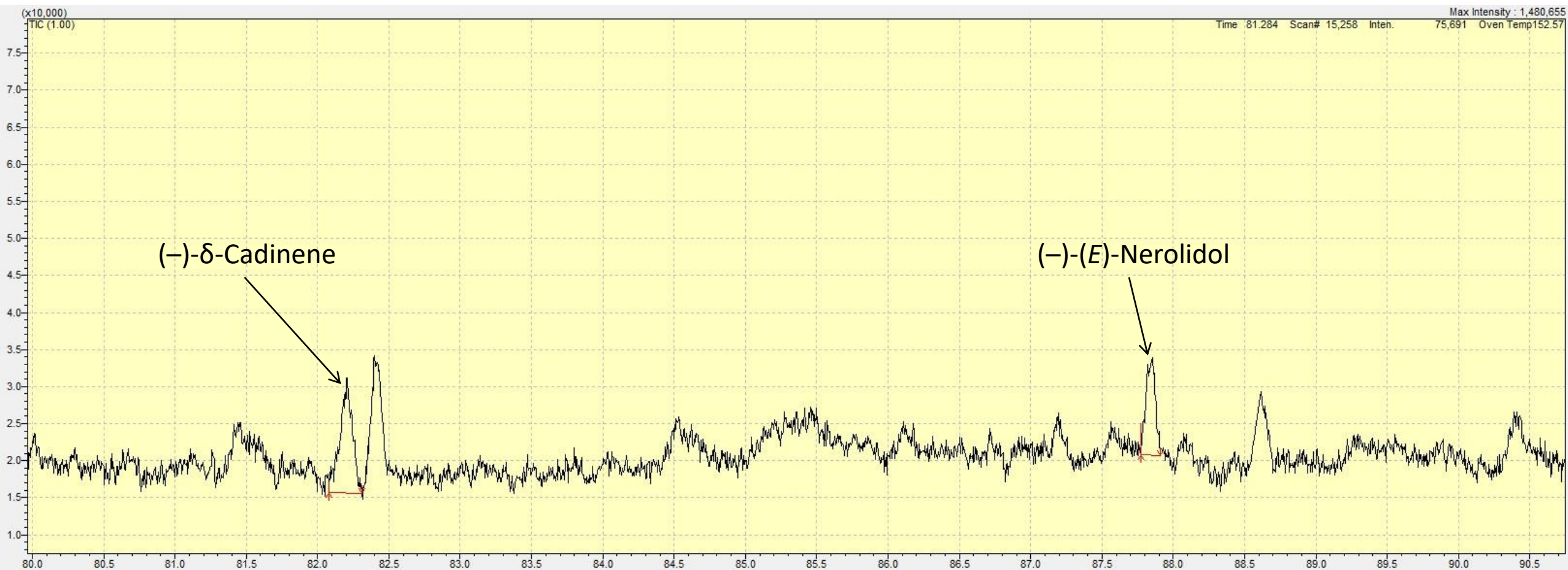

Supplement: Supplementary file 1 [file plants-10-01061-s001.zip › Figure_S5_P_incanum_chiral_GC-MS.pdf]

**Supplementary Figure S6.** Chiral gas chromatogram of *Smallanthus uvedalia* essential oil.

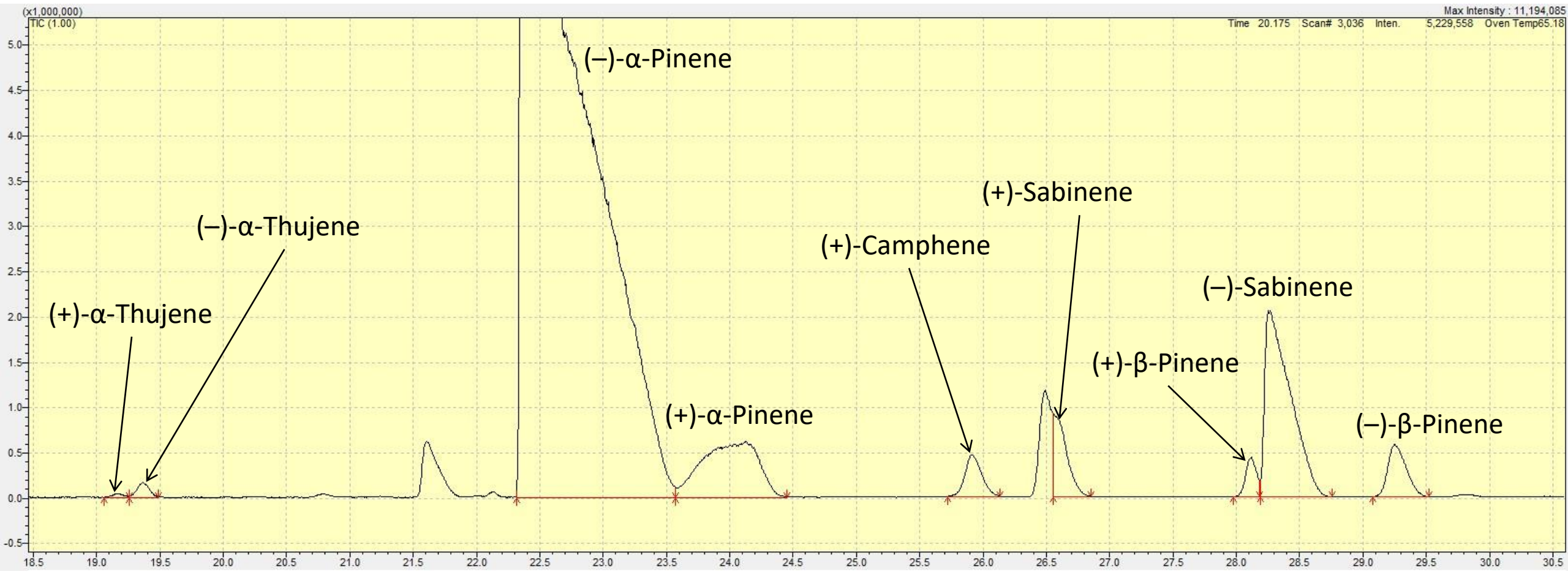

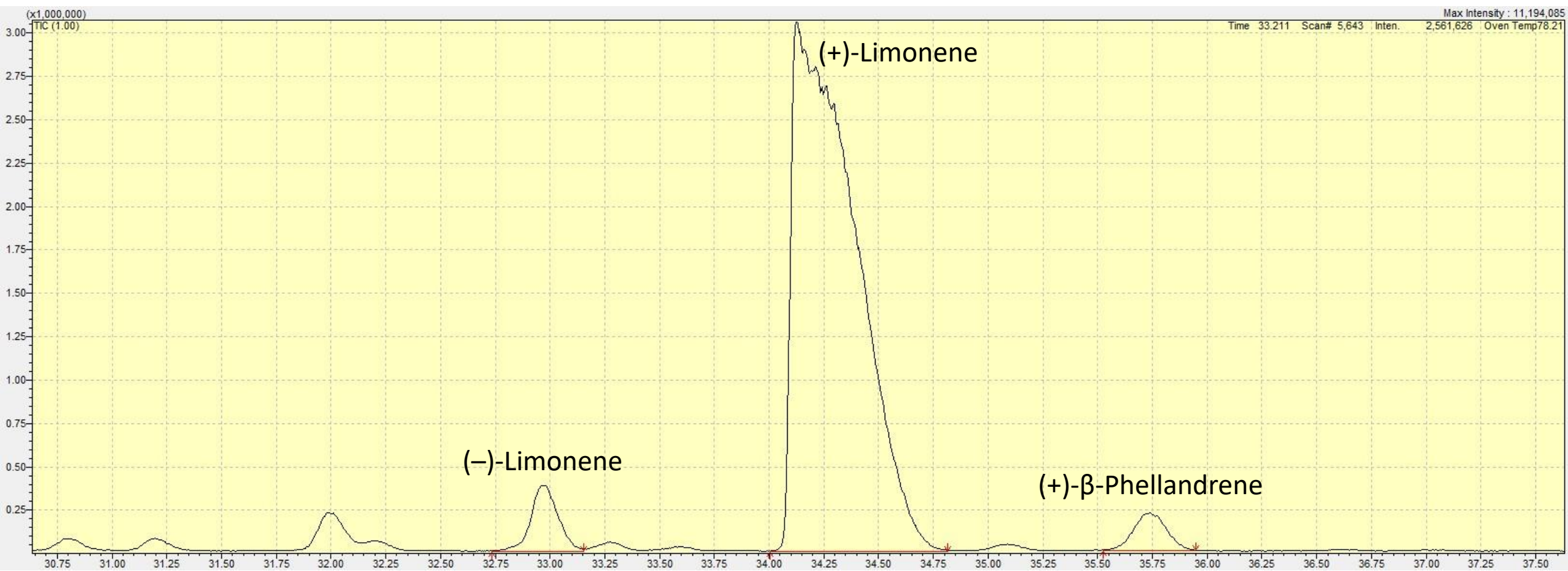

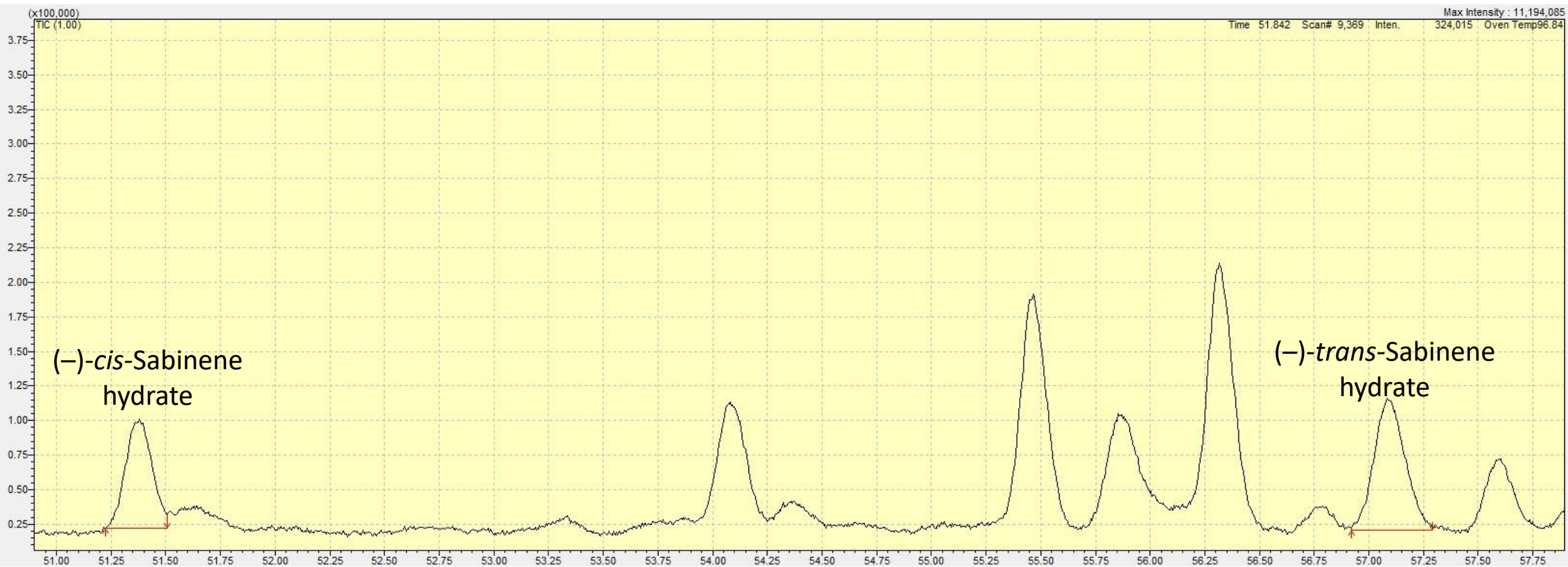

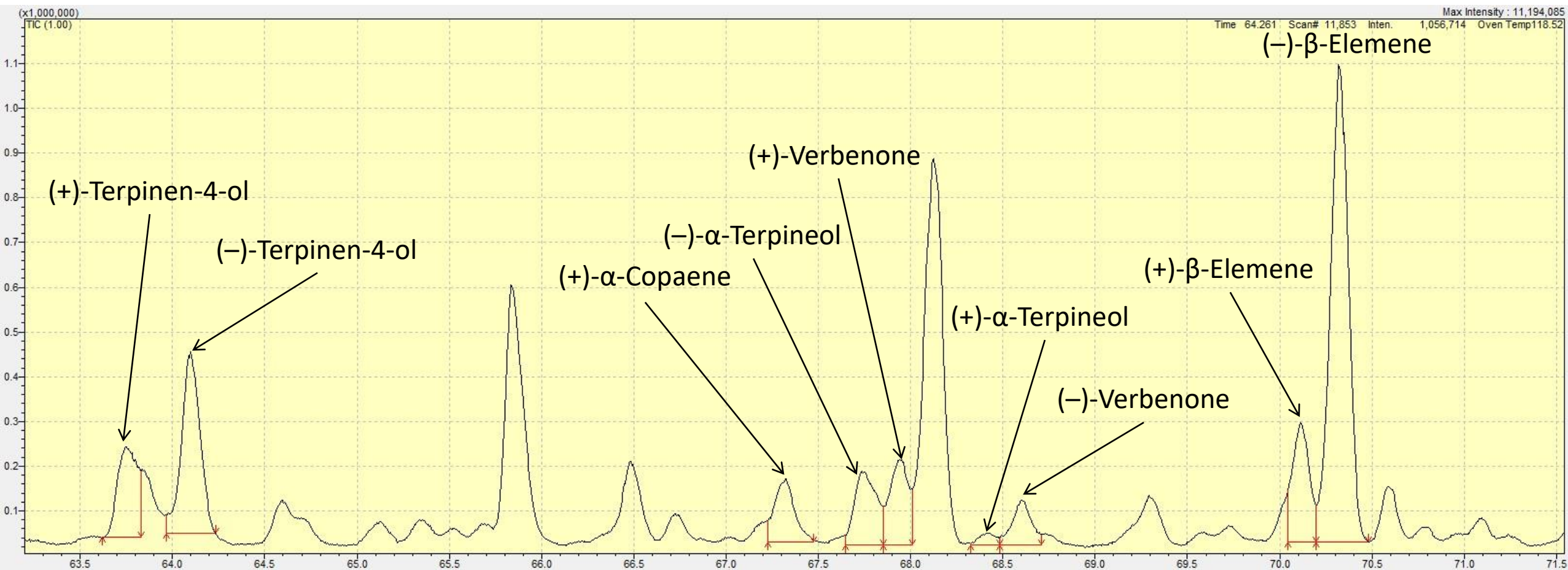

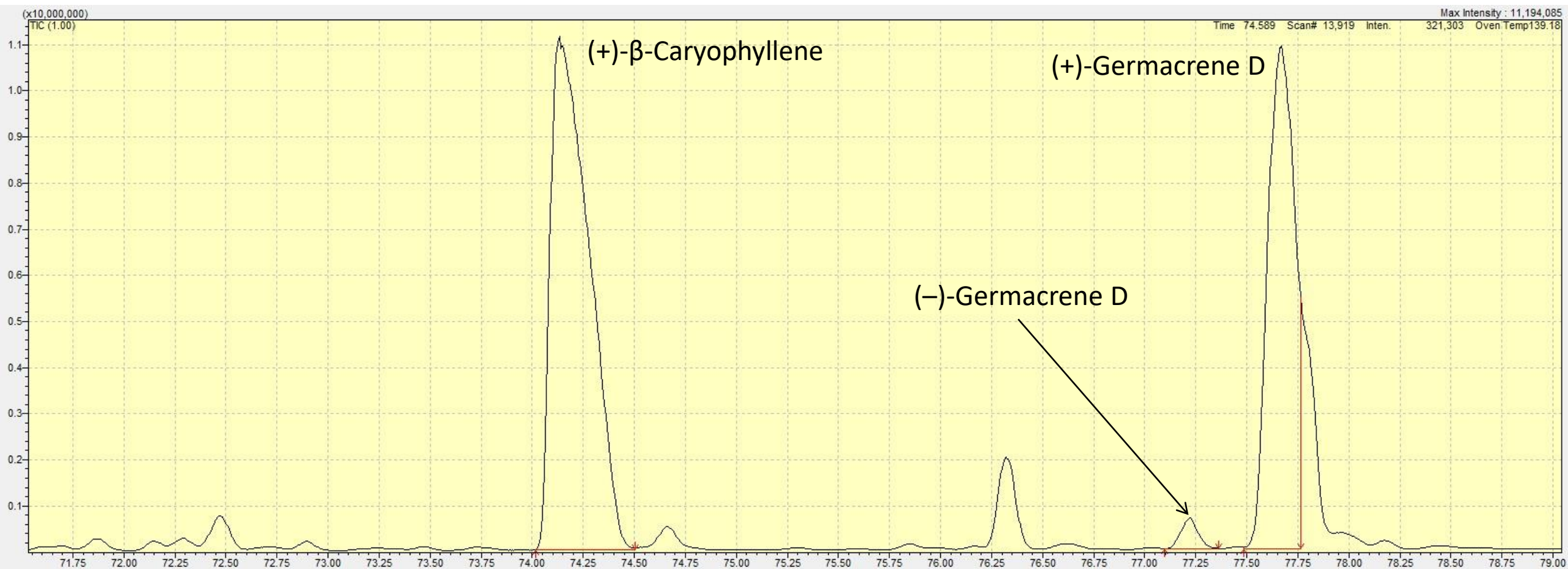

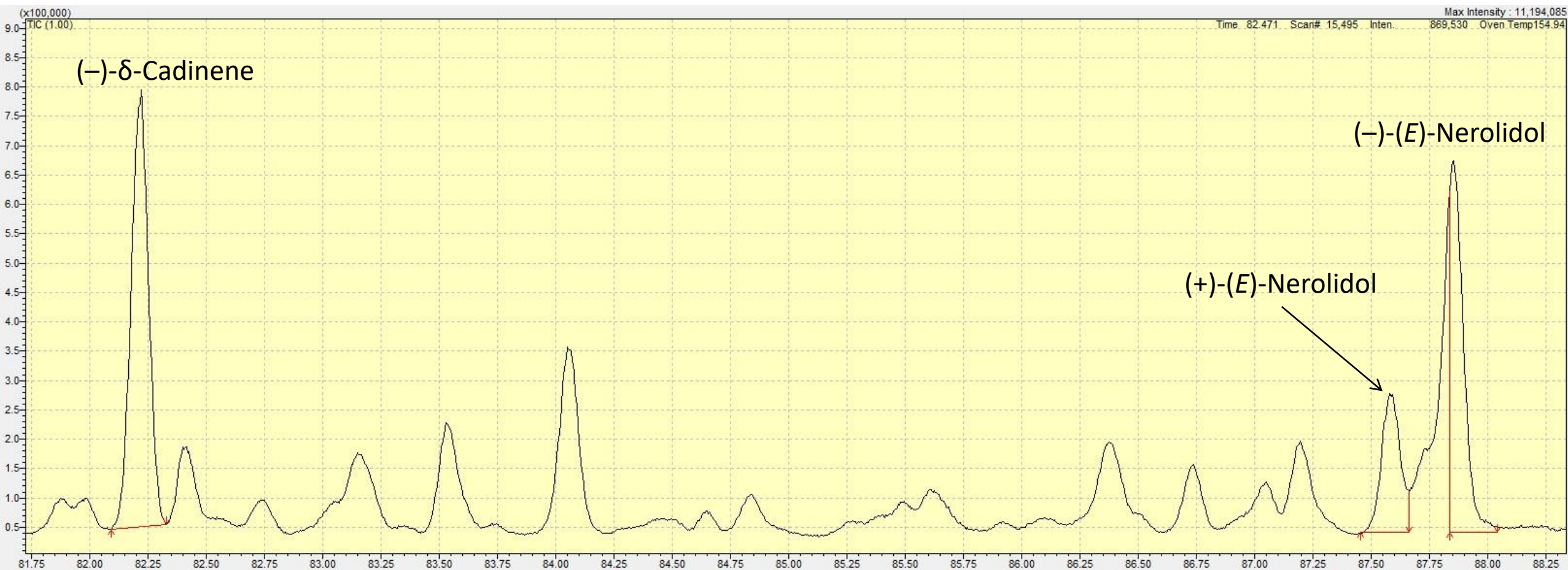

Supplement: Supplementary file 1 [file plants-10-01061-s001.zip › Figure_S6_S_uvedalia_chiral_GC-MS.pdf]

**Supplementary Figure S7.** Chiral gas chromatogram of *Verbena hastata* essential oil.

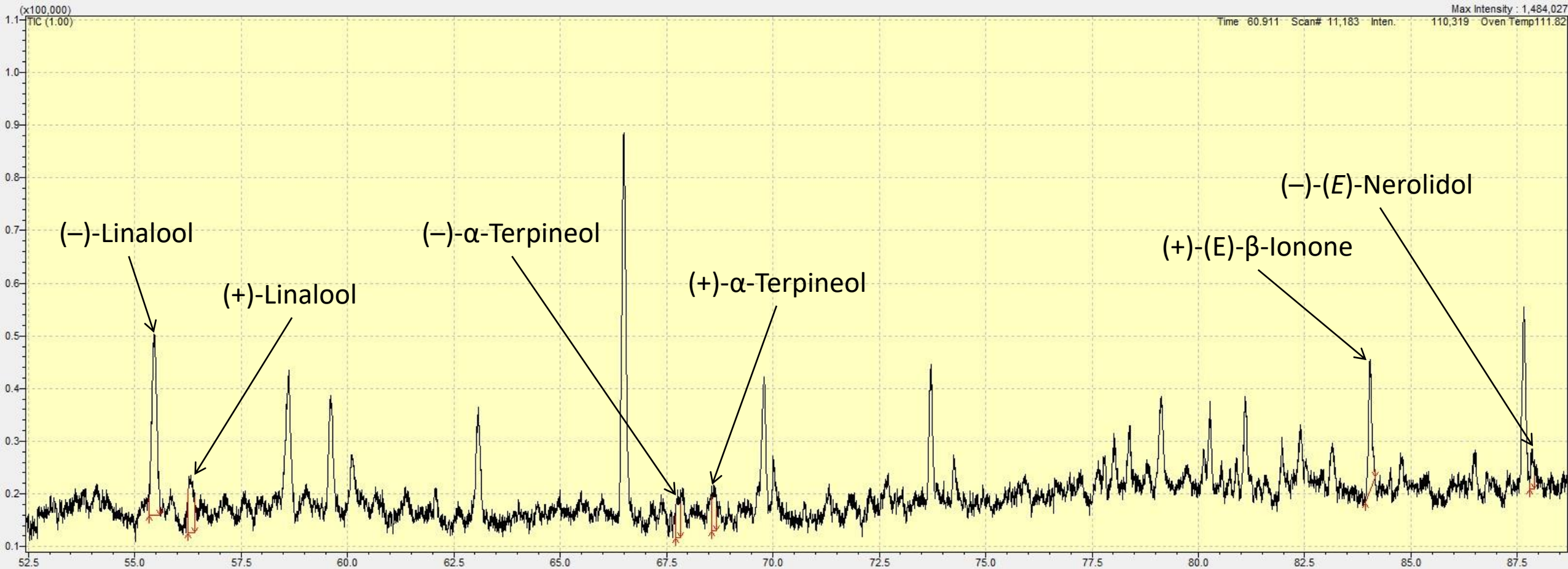

Supplement: Supplementary file 1 [file plants-10-01061-s001.zip › Figure_S7_V_hastata_chiral_GC-MS.pdf]
